# Benchmarking CRISPR-BP34 for point-of-care melioidosis detection in low-income and middle-income countries: a molecular diagnostics study

**DOI:** 10.1016/S2666-5247(23)00378-6

**Published:** 2024-04

**Authors:** Sukripong Pakdeerat, Phumrapee Boonklang, Kesorn Angchagun, Chalita Chomkatekaew, Navaporn Apichaidejudom, Yaowaret Dokket, Areeya Faosap, Gumphol Wongsuwan, Vanaporn Wuthiekanun, Panatda Aramrueung, Phadungkiat Khamnoi, Hathairat Thananchai, Suwattiya Siriboon, Parinya Chamnan, Sharon J Peacock, Nicholas P J Day, Nicholas R Thomson, Chayasith Uttamapinant, Somsakul Pop Wongpalee, Claire Chewapreecha

**Affiliations:** a Mahidol-Oxford Tropical Medicine Research Unit, Faculty of Tropical Medicine, Mahidol University, Bangkok, Thailand; b Department of Clinical Tropical Medicine, Faculty of Tropical Medicine, Mahidol University, Bangkok, Thailand; c Central Laboratory, Sunpasitthiprasong Hospital, Ubon Ratchathani, Thailand; d Department of Infectious Medicine, Sunpasitthiprasong Hospital, Ubon Ratchathani, Thailand; e Cardiometabolic Research Group, Department of Social Medicine, Sunpasitthiprasong Hospital, Ubon Ratchathani, Thailand; f Diagnostic Laboratory, Maharaj Nakorn Chiang Mai Hospital, Chiang Mai, Thailand; g Department of Microbiology, Faculty of Medicine, Chiang Mai University, Chiang Mai, Thailand; h Department of Medicine, University of Cambridge, Cambridge, UK; i Centre for Tropical Medicine and Global Health, Nuffield Department of Medicine, University of Oxford, Oxford, UK; j Parasites and Microbes Programme, Wellcome Sanger Institute, Hinxton, UK; k School of Biomolecular Science and Engineering, Vidyasirimedhi Institute of Science and Technology, Rayong, Thailand

## Abstract

**Background:**

Melioidosis is a neglected but often fatal tropical disease. The disease has broad clinical manifestations, which makes diagnosis challenging and time consuming. To improve diagnosis, we aimed to evaluate the performance of the CRISPR-Cas12a system (CRISPR-BP34) to detect *Burkholderia pseudomallei* DNA across clinical specimens from patients suspected to have melioidosis.

**Methods:**

We conducted a prospective, observational cohort study of adult patients (aged ≥18 years) with melioidosis at Sunpasitthiprasong Hospital, a tertiary care hospital in Thailand. Participants were eligible for inclusion if they had culture-confirmed *B pseudomallei* infection from any clinical samples. Data were collected from patient clinical records and follow-up telephone calls. Routine clinical samples (blood, urine, respiratory secretion, pus, and other body fluids) were collected for culture. We documented time taken for diagnosis, and mortality at day 28 of follow-up. We also performed CRISPR-BP34 detection on clinical specimens collected from 330 patients with suspected melioidosis and compared its performance with the current gold-standard culture-based method. Discordant results were validated by three independent qualitative PCR tests. This study is registered with the Thai Clinical Trial Registry, TCTR20190322003.

**Findings:**

Between Oct 1, 2019, and Dec 31, 2022, 876 patients with culture-confirmed melioidosis were admitted or referred to Sunpasitthiprasong Hospital, 433 of whom were alive at diagnosis and were enrolled in this study. Median time from sample collection to diagnosis by culture was 4·0 days (IQR 3·0–5·0) among all patients with known survival status at day 28, which resulted in delayed treatment. 199 (23%) of 876 patients died before diagnosis and 114 (26%) of 433 patients in follow-up were treated, but died within 28 days of admission. To test the CRISPR-BP34 assay, we enrolled and collected clinical samples from 114 patients with melioidosis and 216 patients without melioidosis between May 26 and Dec 31, 2022. Application of CRISPR-BP34 reduced the median sample-to-diagnosis time to 1·1 days (IQR 0·7–1·5) for blood samples, 2·3 h (IQR 2·3–2·4) for urine, and 3·3 h (3·1–3·4) for respiratory secretion, pus, and other body fluids. The overall sensitivity of CRISPR-BP34 was 93·0% (106 of 114 samples [95% CI 86·6–96·9]) compared with 66·7% (76 of 114 samples [57·2–75·2]) for culture. The overall specificity of CRISPR-BP34 was 96·8% (209 of 216 samples [95% CI 93·4–98·7]), compared with 100% (216 of 216 samples [98·3–100·0]) for culture.

**Interpretation:**

The sensitivity, specificity, speed, and window of clinical intervention offered by CRISPR-BP34 support its prospective use as a point-of-care diagnostic tool for melioidosis. Future development should be focused on scalability and cost reduction.

**Funding:**

Chiang Mai University Thailand and Wellcome Trust UK.

## Introduction

Melioidosis is a neglected tropical disease with a high case fatality (10–50%) even when appropriately treated.^1^ It is estimated to affect 165 000 individuals annually worldwide, of whom 89 000 die from the disease, and the global disease burden is 4·64 million disability-adjusted life-years, 99% of which are accounted for by years of life lost.^2^ The high mortality of melioidosis might be explained by the disease disproportionately affecting rural populations in low-income and middle-income countries,^3^ where poor socioeconomic conditions often result in patients seeking health care when the disease has reached a terminal or critical phase.^4^ Melioidosis is caused by *Burkholderia pseudomallei*, an environmental bacterium in soil and water across the tropical regions of Asia–Pacific,^5^ south Asia,^6^ and southeast Asia.^7^ However, the disease remains largely under-reported due to its non-specific clinical manifestations that are similar to several other diseases. A lack of disease awareness in clinics and communities together with the paucity of diagnostic facilities leads to missed or delayed diagnosis. With early diagnosis and appropriate treatment, the case fatality rate from melioidosis can be decreased to 9% as observed in Australia.^8^

**Research in context**

**Evidence before this study**

We searched PubMed from database inception to Oct 17, 2023, using the terms “melioidosis” AND “diagnosis test,” with no language restrictions and found 210 publications, 40 of which presented clinical evaluations of rapid melioidosis diagnostic tests. Antigen-based diagnostic tests, which detect the presence of *Burkholderia pseudomallei*, reported high specificity (median 98·6% [IQR 94·0–100·0]), but low sensitivity (median 57·1% [IQR 44·3–82·5]). The sensitivity is affected by the often-low concentration of the bacterial antigens in patients' samples, which can vary by specimen type and stage of infection. Antibody-based diagnostic tests that detect host antibodies against *B pseudomallei* typically have satisfactory specificity (median 94·5% [IQR 88·6–96·2]) but poor sensitivity (80·2% [71·0–88·1]). These tests are often affected by variations in antibody responses to *B pseudomallei* and the duration required for antibody production. Furthermore, standardisation remains challenging due to the influence of different serum titres on sensitivity and background of the tests. Likewise, quantitative PCR has high specificity (99·8% [91·6–100·0]), but an observed low sensitivity for melioidosis (77·1% [20·8–97·8]), which is probably due to the low initial bacterial load or the genetic heterogeneity of *B pseudomallei* genomes, or both. Additionally, these studies consistently reported a demand for improved speed and ease of implementation in resource-limited settings where melioidosis is endemic. With the limitations of current diagnostic methods, a culture-confirmed approach with 60% sensitivity, 100% specificity, and a diagnosis time of 2–7 days is still the gold standard for melioidosis diagnosis.

**Added value of this study**

To our knowledge, no study has measured the effect of delayed diagnosis on patients with melioidosis. We assessed the number of deaths occurring before culture-confirmed diagnosis and those after diagnosis but within 28 days after admission, highlighting the urgent need for prompt action. To address this, we developed the CRISPR-BP34 diagnostic test, which uses isothermal amplification of *B pseudomallei* DNA followed by site-specific detection using a CRISPR-associated Cas12a enzyme. We successfully implemented this assay in a resource-limited setting in northeast Thailand, where the disease prevalence is among the highest in the world. The assay achieved a diagnostic sensitivity of 93·0% and specificity of 96·8%, with an estimated limit of detection of 50–250 colony-forming units per mL. Depending on the specimen type, early diagnosis can be achieved within 4 h to 1 day after patient admission. This time to diagnosis is significantly faster than culture, which typically takes several days. Furthermore, the CRISPR-BP34 assay detected low *B pseudomallei* concentrations in haemoculture bottles, which could be missed by culture due to mixed infections, contamination, or other causes.

**Implications of all the available evidence**

The CRISPR-BP34 assay has potential for the management and control of melioidosis, by contributing to the prevention of undiagnosed melioidosis. Its speed and heightened sensitivity enable early diagnosis and treatment, which are crucial for saving patients' lives. Additionally, the minimal setup and user-friendly learning curve make the assay ideal for resource-limited settings.

Clinical specimens from patients with suspected melioidosis are typically screened for the presence of *B pseudomallei* using microbial culture, which has been the gold-standard diagnostic method for the past three decades. This method is imperfect, with a specificity of 100% but a sensitivity of 60%.^9^ *B pseudomallei* exhibits slower growth in laboratory conditions compared with other pathogens.^10^ This delay can lead to the proliferation of other bacteria or fungi within the sample due to mixed infection or contamination, which results in failure to detect *B pseudomallei*. When cultured successfully, *B pseudomallei* colonies can be mistaken for environmental contaminants, necessitating correct identification by a skilled microbiologist. Moreover, the combined time required for both growing and identifying *B pseudomallei* could extend to 7 days, resulting in delays in diagnosis.^9^ Culture-free antigen-based and nucleic acid-based tests such as a lateral flow immunoassay,^11^ an immunofluorescence assay,^12^ PCR,^13^, ^14^, ^15^ or 16S rRNA sequencing,^16^ have been developed for diagnosing melioidosis. However, the variability in bacterial concentrations^17^^,^^18^ across clinical specimens results in low sensitivities of 58·2% (95% CI 34·1–78·9) for the lateral flow immunoassay^19^ and 63·8% (45·6–78·7) for the immunofluorescence assay,^19^ whereas tests that offer higher sensitivity require thermal cyclers or sequencing machines,^16^ which are rarely available in rural settings.

We hypothesised that an improved sensitivity and specificity through the detection of *B pseudomallei* DNA from direct clinical specimens could improve melioidosis diagnosis. One such method is CRISPR-based diagnostics, which involves amplifying the pathogen’s DNA using isothermal recombinase polymerase amplification and then using the sequence-specific recognition of CRISPR-Cas endoribonuclease at the DNA target. This approach has been applied to other bacterial pathogens including *Mycobacterium tuberculosis*^20^ and has been shown to improve diagnosis and treatment responses. We previously described a robust CRISPR-Cas12a-based detection of genomic DNA of *B pseudomallei* in vitro.^21^ Here, we addressed the issues surrounding delayed diagnosis of melioidosis. We aimed to establish a diagnostic protocol for our recently developed CRISPR-Cas12a system^21^ (hereafter termed CRISPR-BP34), and to determine its sensitivity and specificity for diagnostic uses.

## Methods

### Study design and participants

Two related studies were conducted and are reported here. Study 1 assessed the time required for melioidosis diagnosis through culture and patient outcomes, whereas study 2 evaluated the diagnostic performance of the CRISPR-BP34 assay.

Study 1 was a prospective observational cohort study of adult patients (aged ≥18 years) with melioidosis at Sunpasitthiprasong Hospital, a tertiary care hospital in Ubon Ratchathani, Thailand (appendix 2 pp 4–7). Participants were eligible for inclusion if they had culture-confirmed *B pseudomallei* infection from any clinical samples and were resident in northeast Thailand. Participants with tuberculosis, HIV, or immunosuppressive conditions that might affect the infectious outcomes were excluded. The study received ethical approval from the Ethical Review Board of Sunpasitthiprasong Hospital (015/62C) and the Oxford Tropical Research Ethics Committee (OxTREC 25-19). All participants provided written informed consent.

Study 2 was a diagnostic accuracy study conducted at Sunpasitthiprasong Hospital to evaluate the sensitivity and specificity of the CRISPR-BP34 prototype assay.^21^ Participants were identified through the hospital computer system and were included if they were suspected of having melioidosis and had sufficient leftover clinical samples. The study received ethics approval from the Ethical Review Board of Sunpasitthiprasong Hospital (029/65C) and received ethics exemption from Chiang Mai University (9190/2565) for secondary research use of biospecimens for which the identity of human participants cannot be readily ascertained.

### Procedures

For study 1, all patients with culture-confirmed melioidosis were identified through the hospital computer system. Consent was obtained to collect patient clinical records, including diagnosis duration, the antibiotics prescribed before culture confirmation (appendix 2 pp 6–7), and the patients’ 28-day survival status from admission, which was tracked through follow-up telephone calls. Collected data also included patient demographics, symptoms, the interval between symptom onset and seeking health care, the duration of culture-confirmed diagnosis, and the antibiotics prescribed during the unconfirmed period. Patient sex data were collected from hospital records and patient ethnicity data were self-reported. In routine clinical practice, blood, urine, respiratory secretions or fluid (sputum, tracheal suction, and pleural fluid), and other available body fluid and tissues (pus, limb tissue, and synovial fluid), were consecutively collected for culture from patients suspected of having melioidosis. The standard culture methods used for each specimen type are outlined in appendix 2 (p 6), serving as a reference for evaluating CRISPR-BP34 in study 2. 1 mL of leftover sample from the culture was obtained from the hospital microbiology laboratory and stored at –20°C for CRISPR-BP34 screening. Once the culture results arrived, a head-to-head CRISPR-BP34 assay was performed (appendix 2 pp 9–11, 13–19).

Different types of clinical samples require sample-specific preparation due to the variable amounts of target bacterial cells,^17^ host cells, and inhibitors present in each sample. Further details of how the assay was performed on each sample type are described in appendix 2 (pp 9–11). Briefly, for each clinical sample, human cells were first depleted using a simple buffer system to selectively lyse human cells, leaving a pellet of bacterial cells. Bacterial genomic DNA was then extracted from the pellet using either hot alkaline lysis or a GeneJet spin column (ThermoFisher Scientific, Waltham, MA, USA), depending on the pellet size. *B pseudomallei* DNA was amplified in a recombinase polymerase amplification reaction (TwistDX, Maidenhead, UK), and the resulting amplicons were added into a 50 μL CRISPR reaction, comprising CRISPR RNA (crBP34), LbCas12a protein, and FAM-biotin probes (IDT, Singapore; appendix 2 p 8). This reaction was incubated at 37°C for 60 min, after which a HybriDetect lateral flow dipstick (Milenia Biotec, Giessen, Germany) was directly immersed into the reaction and allowed to develop for 5 min before reading by eye. A positive result was defined as the appearance of an upper band (anti-IgG) on the dipstick. The assay was performed in a batch of ten samples with each batch consisting of culture-positive and culture-negative samples to avoid batch effect. For all batches, *B pseudomallei*-positive sample and distilled water were also used as positive and negative controls, respectively. The sample-to-result time was recorded. The CRISPR-BP34 results were interpreted by three different readers who were masked to the patient’s disease status and culture results.

Discordant results between culture and the CRISPR-BP34 assay were tested by quantitative PCR (qPCR) using three primer sets (primer sets are listed in appendix 2 pp 11, 25–26). Owing to a large discrepancy in reported diagnostic sensitivity of PCR primers^19^ and a high sequence diversity of *B pseudomallei*, the use of primer combinations ensured an increased coverage of the detection. The qPCR cycle threshold (Ct) values were recorded and used as a proxy for bacterial loads.

To estimate the potential range of the limit of detection of the CRISPR-BP34 assay, we also conducted in vitro experiments by inoculating genetically modified *Escherichia coli* that harboured a target DNA of the CRISPR-BP34 in its genome (appendix 2 p 8) into blood and urine samples from a single healthy donor at different concentrations (0, 10, 50, 100, 250, 500, 2500, and 5000 colony-forming units [CFU] per mL). Four to five biological replicates were performed for each experiment. CRISPR-BP34 detection was performed on blood and urine as described in appendix 2 (pp 9–11). Blood and urine were selected to represent the most common types of clinical specimens processed from patients with suspected melioidosis. Additional information on how using modified *E coli* as a surrogate for *B pseudomallei* might affect the experiment can be found in appendix 2 (p 9).

### Outcomes

The primary outcome of study 1 was 28-day mortality in patients with culture-confirmed diagnosis of melioidosis. The study 1 secondary outcomes were the time taken for diagnosis, treatments administered, and the resulting infection outcomes. The primary outcomes of study 2 were the evaluation of CRISPR-BP34’s clinical sensitivity, specificity, and assay time against the gold-standard culture-confirmed diagnosis.

### Statistical analysis

The number of patients with melioidosis admitted to Sunpasitthiprasong Hospital during the study period determined the sample size for study 1. The minimum sample size for study 2 was determined using the formula n=z^2^ × p × (1–p)/d^2^ where z is a 95% CI of 1·96; p is a prevalence of 0·5; and d represents a margin of error of 0·1.^22^ At least 96 patients with melioidosis and 96 patients without melioidosis were required for the accuracy evaluation of the CRISPR-BP34 diagnostic test in study 2. Patient demographic data were summarised using medians, IQRs, and proportions. To evaluate 28-day mortality associated with each factor, Kaplan–Meier survival curves, alongside univariable and multivariable Cox proportional hazard regression were employed with hazard ratios (HRs) and their 95% CIs reported. Factors associated with melioidosis mortality were chosen from literature reviews.^1^^,^^2^ These factors were patient demographics (age, sex, and self-reported ethnicity), symptoms (presence or absence of specific symptoms), time from symptom onset to primary health care and diagnosis (categorised as ≤7 days, 8–14 days, 15–21 days, or >21 days), and antibiotic prescription (presence or absence). Sensitivity and specificity were separately calculated for culture and CRISPR-BP34 and categorised by sample type (blood; urine; respiratory secretion; and pus, tissue, and other body fluids) and by total specimens. If a patient had multiple samples, only the earliest sample was used for these calculations. Exact 95% CIs were estimated using a binomial assumption. McNemar’s test was used to compare the performance of culture and CRISPR assay using paired data. For comparison of variables observed from the assays with non-parametric distribution, Wilcoxon signed-rank tests were used. All tests were two-sided, with a significance level of 0·05. R (version 4.3.1) was used for all analyses and data visualisation. Study 1 is registered with the Thai Clinical Trial Registry, TCTR20190322003.

### Role of the funding source

The funders of the study had no role in study design, data collection, data analysis, data interpretation, or writing of the report.

## Results

**Figure 1:**
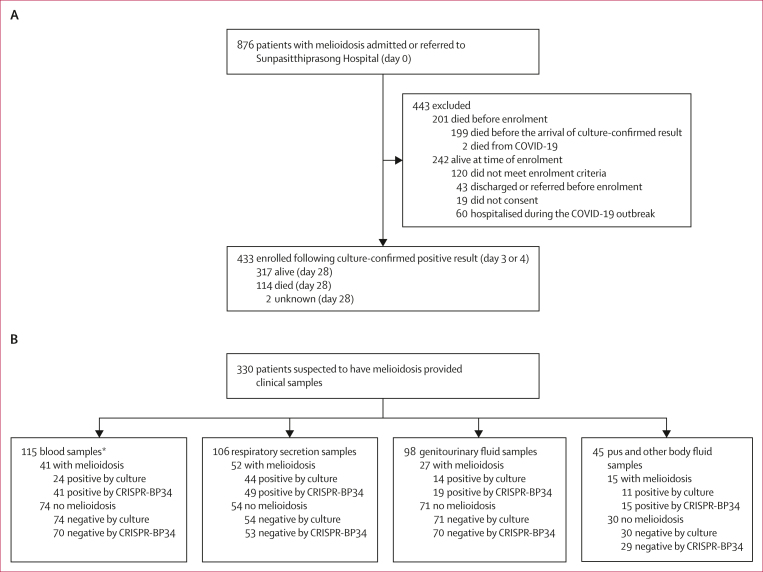
Study profile (A) Study profile for study 1, showing the enrolment and mortality of all patients with melioidosis admitted or referred to Sunpasitthiprasong Hospital during the study period. (B) Study profile for study 2. Some patients had multiple sample types collected so sample numbers do not total 330. ∗Enriched by haemoculture.

Between Oct 1, 2019, and Dec 31, 2022, 876 patients with culture-confirmed melioidosis were admitted or referred to Sunpasitthiprasong Hospital, of whom 199 died before the completion of culture results (figure 1A). Of the 433 patients who were alive when culture results were ready and were enrolled in this study, 431 had known infectious outcomes. Among these patients, 114 (26%) died within 28 days after first presentation to Sunpasitthiprasong Hospital. The minimum fatality of 313 (36%) of 876 patients based on the combined data is consistent with the 35% mortality reported for melioidosis in Thailand.^22^ Patients lived a median 65 km (IQR 40–100) from the hospital, with the majority being agricultural workers (table). Patients with melioidosis had diverse clinical manifestations, with persistent fever being the most common symptom (table; appendix 2 pp 5, 23). Patients had symptoms for a median of 7·0 days (IQR 3·0–14·0) before seeking medical care at local or central health-care centres, which subsequently referred them to Sunpasitthiprasong Hospital within 1·0 days (0·0–4·0). Multiple samples were collected from patients for culture-based diagnosis at their initial admission or referral and during their stay at Sunpasitthiprasong Hospital as clinically indicated. The median duration between the first sample collection and the first positive culture result was 4·0 days (3·0–5·0), at which point melioidosis diagnosis was confirmed. Time from symptom onset to diagnosis was a median of 16·0 days (9·0–27·0), and was shorter in patients who died (12·0 days [7·0–19·0]) than in patients who were alive at day 28 (18·0 days [10·0–31·0]; table; appendix 2 p 20).

**Table:** Baseline demographics for patients enrolled in study 1 who were alive at completion of culture results

	All participants with known survival status (n=431)	Alive at day 28 (n=317)	Died by day 28 (n=114)
Age, years	53 (45–61)	53 (45–61)	55 (45–61)
Age groups, years			
18–30	22 (5%)	15 (5%)	7 (6%)
31–50	153 (35%)	118 (37%)	35 (31%)
51–70	217 (50%)	162 (51%)	55 (48%)
>70	39 (9%)	22 (7%)	17 (15%)
Sex			
Female	127 (29%)	96 (30%)	31 (27%)
Male	304 (71%)	221 (70%)	83 (73%)
Self-reported ethnicity			
Thai	429 (>99%)	317 (100%)	112 (98%)
Laos	2 (<1%)	0	2 (2%)
Occupation			
Agriculture	271 (63%)	201 (63%)	70 (61%)
Private sector	76 (18%)	55 (17%)	21 (18%)
Homemaker	53 (12%)	39 (12%)	14 (12%)
Monk	7 (2%)	5 (2%)	2 (2%)
Police and military	6 (1%)	4 (1%)	2 (2%)
Distance from patient's home to Sunpasitthiprasong Hospital, km	65 (40–100)	65 (40–100)	70 (40–100)
Symptoms			
Fever	277 (64%)	203 (64%)	74 (65%)
Cough	138 (32%)	95 (30%)	43 (38%)
Dyspnoea	69 (16%)	47 (15%)	22 (19%)
Gastrointestinal disturbance	98 (23%)	65 (21%)	33 (29%)
Joint pain	29 (7%)	25 (8%)	4 (4%)
Muscle pain	57 (13%)	45 (14%)	12 (11%)
Local swelling, mass, or abscess	39 (9%)	34 (11%)	5 (4%)
Weight loss	21 (5%)	20 (6%)	1 (1%)
Time to melioidosis diagnosis, days			
Time from symptom onset to approaching health care	7·0 (3·0–14·0)	9·0 (4·0–20·0)	6·5 (3·0–11·8)
Time for referral	1·0 (0·0–4·0)	1·0 (0·0–4·0)	1·0 (0·0–3·0)
Time from sample to culture diagnosis	4·0 (3·0–5·0)	4·0 (3·0–6·0)	3·0 (2·0–5·0)
Time from symptom onset to culture diagnosis	16·0 (9·0–27·0)	18·0 (10·0–31·0)	12·0 (7·0–19·0)
Antibiotic prescription on first presentation to Sunpasitthiprasong Hospital			
Recommended empirical treatment			
Ceftazidime monotherapy	49 (11%)	44 (14%)	5 (4%)
Carbapenem monotherapy	54 (13%)	37 (12%)	17 (15%)
Ceftazidime or carbapenem or both	111 (26%)	87 (27%)	24 (21%)
Ceftazidime or carbapenem but in combination with other antibiotics	305 (71%)	222 (70%)	83 (73%)
Other antibiotics prescribed on first presentation			
Ceftriaxone monotherapy or in combination with other antibiotics	98 (23%)	70 (22%)	28 (25%)
Penicillin monotherapy or in combination with other antibiotics	46 (11%)	36 (11%)	10 (9%)
Lincosamide monotherapy or in combination with other antibiotics	111 (26%)	77 (24%)	34 (30%)
Macrolide monotherapy or in combination with other antibiotics	89 (21%)	59 (19%)	30 (26%)
Nitroimidazole monotherapy or in combination with other antibiotics	41 (10%)	27 (9%)	14 (12%)

Data are median (IQR) or n (%). Symptoms are not mutually exclusive, with patients often presenting with one or more symptoms.

**Figure 2:**
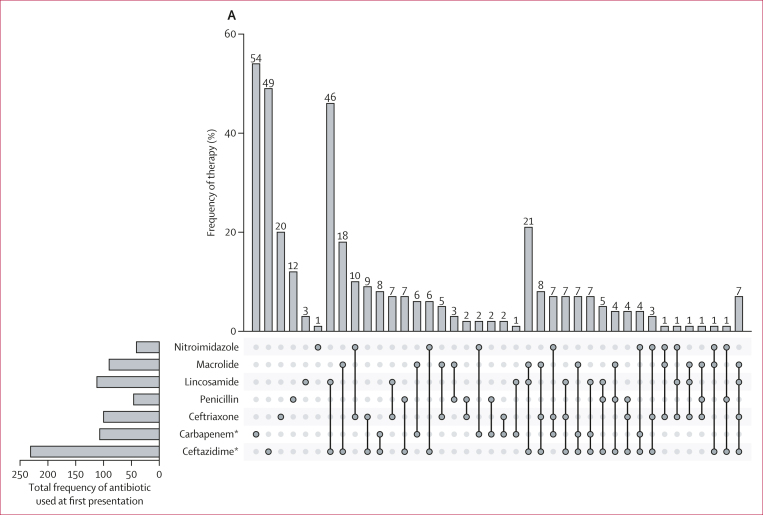

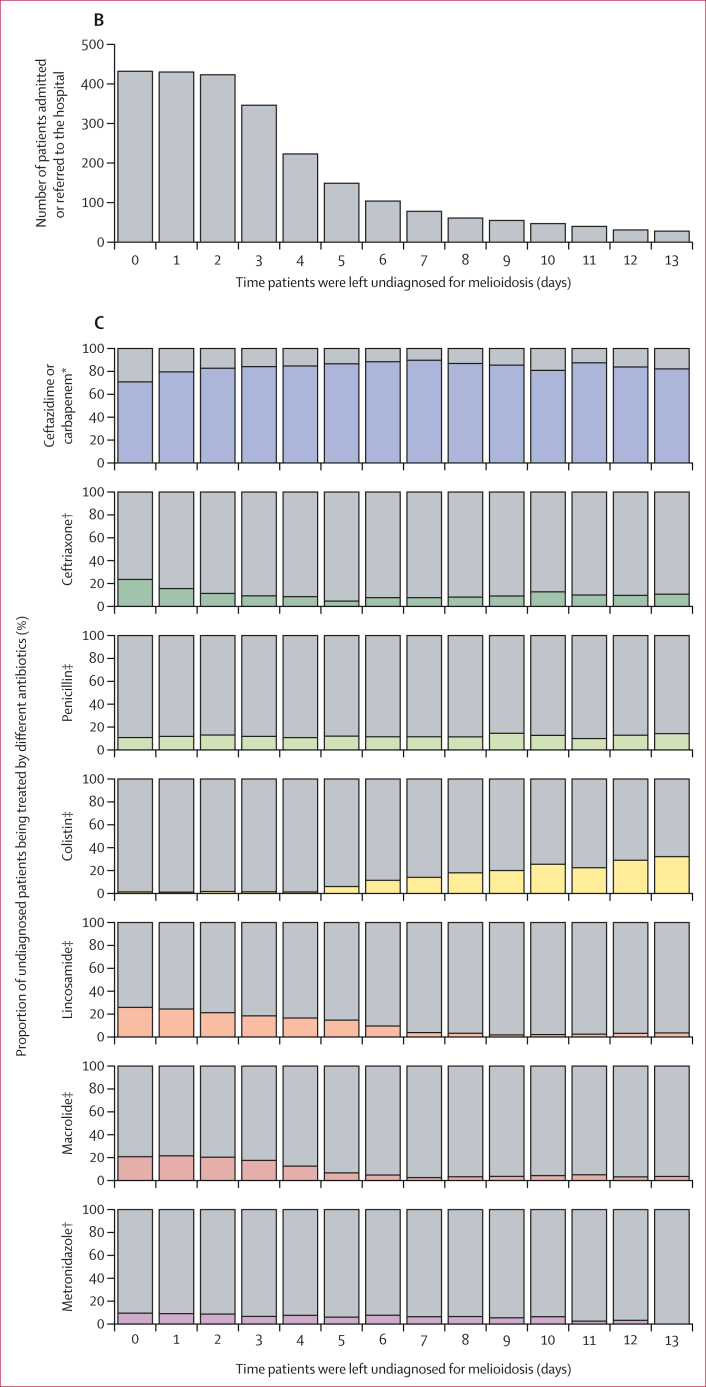
Effect of delayed diagnosis on melioidosis treatments and outcomes (A) An UpSet plot of the antibiotic prescribed when patients first presented to Sunpasitthiprasong Hospital, either as a monotherapy or combination therapy. Numbers above columns indicate the number of patients receiving each therapy. (B) Number of patients who remained undiagnosed on each day after their admission or referral to Sunpasitthiprasong Hospital. Day 0 indicates the day of admission or referral to the hospital. The majority of patients received culture-confirmed diagnosis on day 4. (C) Antibiotics prescribed to patients who remained undiagnosed for melioidosis each day after their admission or referral date (day 0). ∗Thai guidelines recommend ceftazidime or carbapenem monotherapy for melioidosis treatment. †Has low effectiveness against melioidosis. ‡*Burkholderia pseudomallei* is resistant to this antibiotic.

In Thailand, patients suspected of having melioidosis are recommended to receive empirical treatment with intravenous ceftazidime or carbapenem antibiotics, such as meropenem or imipenem, for initial intensive monotherapy.^23^ Antibiotic administration data were available for 433 patients, of whom 49 (11%) received ceftazidime monotherapy and 54 (12%) received carbapenem monotherapy on their first presentation to Sunpasitthiprasong Hospital (figure 2A; table; appendix 2 p 20). 341 (79%) patients received other treatments. These included antibiotics known to be ineffective in treating melioidosis such as lincosamides,^24^ macrolides, penicillin with first-generation and second-generation cephalosporin,^25^, ^26^, ^27^ and ineffective third-generation cephalosporins such as ceftriaxone, either as monotherapy, in combination with each other, or in combination with ceftazidime or carbapenem (figure 2B, C).^28^ A lower mortality (five [10%] of 49) was observed in patients who received ceftazidime monotherapy than in patients who received other types of treatments (109 [29%] of 382, multivariable analysis HR 0·36 [95% CI 0·15–0·89]; Cox regression p=0·029; appendix 2 pp 27–28). No other treatments or factors showed significant association with 28-day mortality in the study population in the univariable and multivariable Cox regression analysis.

**Figure 3:**
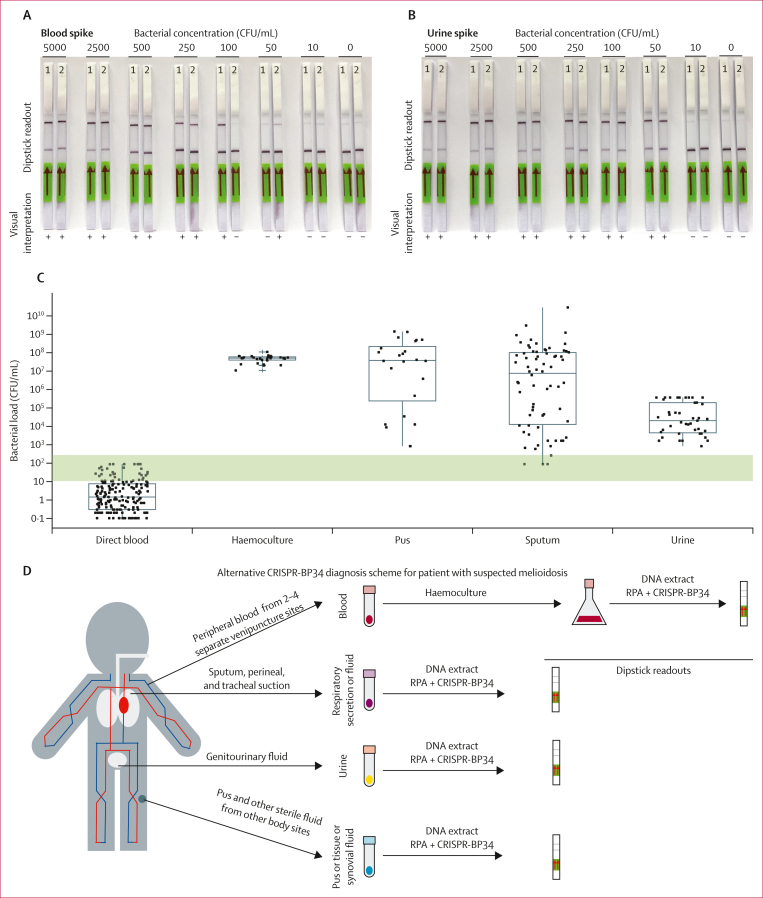
Limit of detection of the CRISPR-BP34 test and its proposed diagnostic pipeline Lateral flow dipsticks showing the limit of detection of the CRISPR-BP34 test in blood (A) and urine (B) samples. The lowest bacterial concentrations detected in blood were observed at 50, 100, 100 and 250 CFU/mL (four biological replicates), while in urine, the lowest concentrations were reported at 10, 10, 10, 50 and 50 CFU/mL (five biological replicates). The dipsticks are shown as two biological replicates. (C) The number of *Burkholderia pseudomallei* cells recovered from different specimen types showing median, IQR, and range. Green bar indicates the range of the CRISPR-BP34 test’s limit of detection. (D) Overview of the CRISPR-BP34 diagnostic pipeline based on clinical samples including blood, genitourinary fluid, respiratory secretion, pus, and other body fluids that were routinely collected from patients suspected of having melioidosis. Bacterial pathogens in blood are enriched for growth to the detectable threshold (250 CFU/mL) through haemoculture. CFU=colony-forming units. RPA=recombinase polymerase amplification.

In study 2, we conducted spiking experiments to establish the limit of detection of CRISPR-BP34. We estimated the limit of detection to be 250 CFU/mL in blood samples and 50 CFU/mL in urine samples (figure 3A, B). To assess whether the CRISPR-BP34 test is sufficiently sensitive to detect *B pseudomallei* across various clinical samples, we enumerated the numbers of bacterial cells recovered from diverse specimen types (figure 3C). Our results showed that the number of *B pseudomallei* cells present in most common specimens such as urine (median 2·6 × 10^4^ CFU/mL [IQR 5⋅5 × 10^3^ to 2⋅6 × 10^5^]), sputum (median 8·8 × 10^7^ CFU/mL [IQR 1⋅7 × 10^4^ to 1⋅6 × 10^8^]), pus and other body fluids (median 5·4 × 10^7^ CFU/mL [IQR 4⋅8 × 10^5^ to 3⋅8 × 10^8^]) were greater than the CRISPR-BP34’s limit of detection. Because direct blood samples had a lower *B pseudomallei* concentration (median 1·5 CFU/mL [IQR 0⋅3 to 8⋅1]) than the limit of detection, we substituted direct blood samples with haemoculture-positive samples (median 7·3 × 10^7^ CFU/mL [IQR 3·0 × 10^7^ to 1·1 × 10^8^]) to ensure sufficient bacterial concentration. Haemoculture-positive samples are blood samples cultured for a period ranging from 2 h to 5 days to enhance bacterial growth, but the bacterial identity remains unknown (figure 3D).

**Figure 4:**
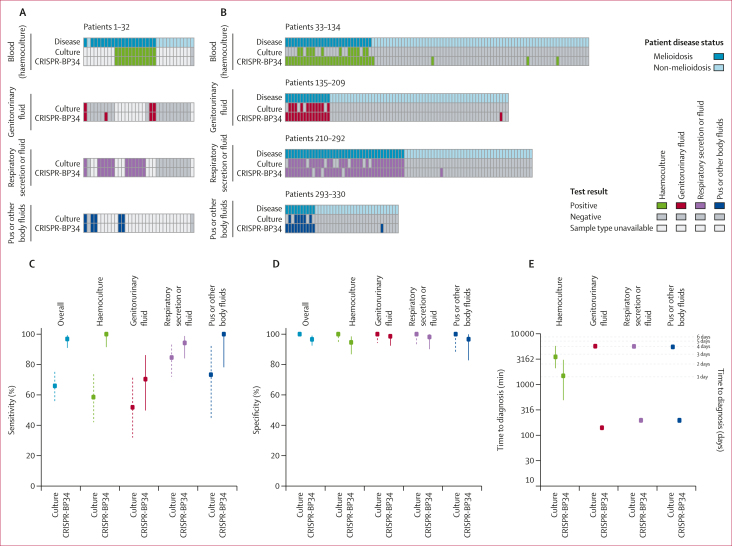
Sensitivity and specificity of the culture and the CRISPR-BP34 approaches (A) Combined panels of diagnostic results from the 32 patients with more than one specimen type. (B) Findings from 298 patients with a single specimen type. In panels A and B, for patients with multiple samples per each specimen type, only the first sample is presented; and each column corresponds to data from each patient with disease status marked as melioidosis or non-melioidosis. (C) Sensitivity of culture compared with CRISPR-BP34. (D) Specificity of culture compared with CRISPR-BP34. (E) Sample-to-diagnosis time of culture compared with CRISPR-BP34. For C–E, dots indicate the actual values, with the 95% CI represented by dashed lines (culture) and solid lines (CRISPR-BP34).

To test the hypothesis that the CRISPR-BP34 assay would be more sensitive and faster than the culture-confirmed approach, between May 26 and Dec 31, 2022, we enrolled and collected clinical samples from 114 patients with melioidosis and 216 patients without melioidosis (figure 1B). 54 participants with melioidosis were also enrolled in study 1. 20 patients with melioidosis and 12 without melioidosis had samples collected across multiple specimen types (figure 4A); the other participants (94 patients with melioidosis and 204 without melioidosis) had a single sample type collected (figure 4B). Using the first sample available from each patient, we estimated the overall diagnostic sensitivity and specificity of culture and CRISPR-BP34. Our findings showed an overall sensitivity of CRISPR-BP34 of 93·0% (106 of 114 samples [95% CI 86·6–96·9]), higher than the sensitivity of culture at 66·7% (76 of 114 samples [57·2–75·2]; figure 4C; appendix 2 p 29). The overall specificity of the CRISPR-BP34 was 96·8% (209 of 216 samples [95% CI 93·4–98·7]), compared with 100% (216 of 216 samples [98·3–100·0]) for culture (figure 4D; appendix 2 p 29). Sensitivity for individual sample types was generally higher for CRISPR-BP34 than for culture (figure 4C; appendix 2 p 29). Specificity for individual sample types was equivalent or slightly lower for CRISPR-BP34 than for culture (figure 4D; appendix 2 p 29). A McNemar’s test further confirmed a significant difference in performance between the CRISPR-BP34 test and culture for total specimens (sensitivity and specificity combined p<0·0001; appendix 2 p 29).

The CRISPR-BP34 assay provided faster results and significantly reduced the turnaround time for all sample types compared with culture (figure 4E, Wilcoxon test p value <0·0001 for all sample types). For culture-positive samples, the median sample-to-result time was 2·5 days (IQR 1·8–3·3) for haemoculture, and 3·9 days (IQR 3·7–4·1) for urine, respiratory secretion, and fluids, as well as other body fluids and tissues. By contrast, the median time from sample collection to positive result for the CRISPR-BP34 assay was 1·1 days (IQR 0·7–1·5) for blood (which required haemoculture), 2·3 h (IQR 2·3–2·4) for urine, and 3·3 h (3·1–3·4) for respiratory secretion and fluids, as well as other body fluids and tissues.

Among 114 patients with melioidosis in study 2, 20 had more than one sample type and multiple samples collected over time for each type (figure 4A; appendix 2 p 21), providing an opportunity to investigate how each patient was diagnosed and treated in real-world scenarios. Early specimens were collected on the first or within a few days of admission or referral to aid disease diagnosis; later specimens were taken at intervals of 3, 5, or 7 days after antibiotic prescription to assess response to the treatment (note that late specimens were not included in this study’s analysis). Nine (45%) of 20 early specimens collected from patients later confirmed to have melioidosis yielded negative culture results. This could be attributed to either the concentration of *B pseudomallei* being below culture’s limit of detection or the samples being contaminated with other fast-growing bacterial species (appendix 2 p 21). One striking example was a haemoculture bottle from a patient with melioidosis that was contaminated with coagulase-negative *Staphylococci*, a skin commensal (appendix 2 p 21). However, the CRISPR-BP34 assay showed a positive *B pseudomallei* result from this contaminated blood bottle, consistent with subsequent qPCR experiments confirming the presence of *B pseudomallei* DNA and with the patient’s final diagnosis. We observed a high Ct value of *B pseudomallei* in the contaminated haemoculture bottle and other contaminated samples (median 32·3 [IQR 30·5–35·9]), compared with median Ct values detected in *B pseudomallei*-positive haemoculture (14·0 [IQR 13·4–14·9]; appendix 2 p 22), which suggests that the *B pseudomallei* population was outcompeted by contaminant species. Regardless, for all patients being followed up, CRISPR-BP34 provided earlier identification of *B pseudomallei* than culture (appendix 2 p 21).

## Discussion

Our study showed that diagnosis using the gold-standard culture method required 3–4 days, with observed delayed treatment and patient deaths before and after culture diagnosis. Implementing CRISPR-BP34 could potentially reduce sample-to-diagnosis time to approximately 1 day for blood samples, and less than 4 h for urine, respiratory secretions, pus, and other body fluids. CRISPR-BP34 showed greater sensitivity than culture with a similar specificity. However, our study has limitations. Notably, severely ill patients died before we could reach them, thereby leading to the number of deaths being underestimated. Nevertheless, our findings echo the problems^2^ of delayed disease diagnosis and imperfect treatment during uncertain diagnoses, both of which might independently or collectively contribute to fatalities.

To improve the appropriate and timely initiation of melioidosis treatment, we developed a CRISPR-BP34 assay (appendix 2 pp 9–19), tested sample-type specific protocols, and evaluated the test performance. To our knowledge, the estimated limit of detection of the assay at the range of 50–250 CFU/mL is the lowest among reported melioidosis rapid diagnosis tests without requiring extensive equipment such as a qPCR machine or an ultraviolet microscope. Although CRISPR-BP34 could detect *B pseudomallei* at a concentration as low as 50 CFU/mL, the miniscule volume of specimens used by the CRISPR approach means that the test can be skewed by inaccurate pipetting or handling errors. Thus, for direct blood samples, we recommend using CRISPR-BP34 on DNA extracted from haemoculture (enriched media), instead of direct blood samples, to maintain high sensitivity. For common clinical specimens with high bacterial loads (>10³ CFU/mL), such as haemoculture samples, genitourinary fluids, and respiratory secretions, as well as pus and other body fluids, CRISPR-BP34 could be used directly on DNA extracted from these samples. Consequently, CRISPR-BP34 exhibited a high level of sensitivity of 93·0% compared with 66·7% sensitivity for overall samples using culture. This enhanced sensitivity facilitated the detection of a minimal population of *B pseudomallei* in the presence of contamination and competition from rapidly growing bacteria in haemoculture (appendix 2 p 21). Cross-contamination incidents are not uncommon in resource-constrained laboratories such as in rural Thailand, potentially leading to an underestimated incidence of melioidosis.^29^

The CRISPR test specificity was slightly lower than the 100% specificity of culture across all sample types. Some of the false positives could be true but missed diagnoses as a result of current imperfect diagnosis techniques including the culture-confirmed approach^9^ and qPCR^19^ with suboptimal primers. Some false positives might also arise when high copy numbers of genetic materials or recombinase polymerase amplification amplicons were mixed or handled in a confined bench setting, which is sometimes unavoidable in the crowded space of resource-limited laboratories. Alternatively, the CRISPR-BP34 complex might unintentionally target other DNA sequences, resulting in false positives. However, the latter is likely to be mitigated by the double-layered specificity provided by recombinase polymerase amplification primers and CRISPR RNA, each of which were carefully designed using a genomic database of more than 40 000 bacterial and human DNA.^21^ To ensure a robust test, we provide suggestions for how to minimise the DNA cross-contamination that could generate false positives in appendix 2 (p 11).

Although our findings support the potential of CRISPR-BP34 as a point-of-care diagnostic tool, further technical refinement is needed to enhance user-friendliness, scalability, and cost-effectiveness. Ongoing efforts are directed towards aligning CRISPR-BP34 with WHO’s point-of-care diagnostics guideline^30^ (appendix 2 pp 12, 30). The implementation of CRISPR-BP34 has the potential to facilitate prompt initiation of life-saving treatment (appendix 2 p 21) and has garnered positive feedback from the Thai Ministry of Public Health and regional health authorities. We believe that a robust melioidosis rapid test, designed for resource-constrained settings, could also prove effective in resource-rich environments.

## Data sharing

De-identified participant data that underlie the results reported in the Article will be made available on request. Proposals should be directed to the corresponding author, claire@tropmedres.ac. Proposals will be reviewed on the basis of compliance with the informed consent and scientific merit.

## Declaration of interests

We declare no competing interests.
