# Supplementary material for: Benchmarking CRISPR-BP34 for point-of-care melioidosis detection in low-income and middle-income countries: a molecular diagnostics study
Source: Lancet Microbe. 2024 Apr;5(4):e379–89. doi: 10.1016/S2666-5247(23)00378-6 (PMC10990966; doi:10.1016/S2666-5247(23)00378-6)
Supplement: Supplementary appendix 1 [file mmc1.pdf]

THE LANCET
Microbe

Supplementary appendix 1

This translation in Thai was submitted by the authors and we reproduce it as supplied. It has not been peer reviewed. The *Lancet's* editorial processes have only been applied to the original in English, which should serve as reference for this manuscript.

**ผลงานแปลไทยชิ้นนี้ถูกส่งมาโดยผู้เขียน
และเรานำมาจัดทำซ้ำตามที่จัดเตรียมไว้โดยที่ยังไม่มีการตรวจทานใด ๆ ทั้งนี้
กระบวนการทางบรรณาธิการของ Lancet ครอบคลุมเฉพาะต้นฉบับในภาษาอังกฤษเท่านั้น
ซึ่งควรใช้เป็นข้อมูลอ้างอิงสำหรับต้นฉบับนี้**

Supplement to: Pakdeerat S, Boonklang P, Angchagun K, et al. Benchmarking CRISPR-BP34 for point-of-care melioidosis detection in low-income and middle-income countries: a molecular diagnostics study. *Lancet Microbe* 2024. https://doi.org/10.1016/S2666-5247(23)00378-6

บทสรุปย่อ

ที่มาและความสำคัญ

โรคเมลิออยโดสิสหรือโรคเมลิออยด์ เป็นโรคที่เกิดจากเชื้อแบคทีเรีย *Burkholderia pseudomallei* (เชื้อเมลิออยด์) ที่พบมากในประเทศไทยและประเทศในเขตร้อนชื้น แม้ว่าโรคนี้จะไม่เป็นที่รู้จักมากนักแต่ก็เป็นโรคที่สามารถก่ออันตรายร้ายแรงถึงชีวิต ผู้ป่วยที่เป็นโรคเมลิออยด์นั้นจะมีอาการแสดงได้หลากหลายรูปแบบ ส่งผลให้การวินิจฉัยทางห้องปฏิบัติการที่ถูกต้องทำได้ยากและใช้เวลานาน ด้วยเหตุนี้ คณะผู้วิจัยจึงได้ทำการพัฒนาชุดตรวจจับดีเอ็นเอของเชื้อเมลิออยด์ด้วยระบบ CRISPR-Cas12a (CRISPR-BP34) ในสิ่งส่งตรวจของผู้ป่วยที่สงสัยว่าเป็นโรคเมลิออยด์ โดยมุ่งหวังว่าชุดตรวจที่พัฒนาขึ้นมานี้จะช่วยทำให้การวินิจฉัยโรคมีความถูกต้องและรวดเร็วขึ้น

วิธีดำเนินการวิจัย

คณะผู้วิจัยทำการศึกษาในผู้ป่วยโรคเมลิออยด์ที่เข้ารับการรักษาที่โรงพยาบาลสรรพสิทธิประสงค์ จังหวัดอุบลราชธานี ประเทศไทย โดยผู้ป่วยที่เข้าร่วมโครงการต้องมีอายุ 18 ปีขึ้นไป และได้รับการยืนยันว่ามีการติดเชื้อเมลิออยด์ด้วยวิธีการเพาะเชื้อจากตัวอย่างตรวจ คณะผู้วิจัยทำการเก็บข้อมูลผู้ป่วยจากทะเบียนประวัติและติดตามซักถามผ่านทางโทรศัพท์ เก็บสิ่งส่งตรวจประเภทต่างๆ ได้แก่ เลือด ปัสสาวะ สารคัดหลั่งจากระบบทางเดินหายใจ น้ำหนอง และสารคัดหลั่งอื่นสำหรับการเพาะเชื้อ รวมถึงเก็บข้อมูลระยะเวลาที่ใช้วินิจฉัยด้วยวิธีการเพาะเชื้อ และตรวจสอบการเสียชีวิตของผู้ป่วยภายในระยะเวลา 28 วัน นอกจากนี้คณะผู้วิจัยได้ทดลองใช้ชุดตรวจ CRISPR-BP34 กับสิ่งส่งตรวจจากผู้ป่วยที่สงสัยว่าติดเชื้อเมลิออยด์ จำนวน 330 ราย และเปรียบเทียบประสิทธิภาพการทำงานของ CRISPR-BP34 กับวิธีการเพาะเชื้อ ในกรณีที่ผลการตรวจจากทั้ง 2 วิธีดังกล่าวไม่สอดคล้องกัน คณะผู้วิจัยได้ใช้เทคนิค quantitative PCR (qPCR) เพื่อทำการยืนยันผล การศึกษานี้ได้ลงทะเบียนกับสำนักทะเบียนงานวิจัยแบบทดลองทางคลินิกของประเทศไทย (Thai Clinical Trial Registry) หมายเลขโครงการ TCTR20190322003

ผลการวิจัย

จากการวิเคราะห์ข้อมูลผู้ป่วยที่เข้ารับการรักษาหรือส่งต่อมายังโรงพยาบาลสรรพสิทธิประสงค์ระหว่างวันที่ 1 ตุลาคม พ.ศ. 2562 ถึง 31 ธันวาคม พ.ศ. 2565 พบว่ามีผู้ป่วยจำนวน 876 รายที่มีผลการเพาะเชื้อเมลิออยด์เป็นบวก ในจำนวนนี้มีผู้ป่วยจำนวน 433 รายที่ยังมีชีวิตอยู่ในขณะที่ได้รับผลการเพาะเชื้อและเข้าร่วมโครงการวิจัย ทั้งนี้ค่าเฉลี่ยของระยะเวลาที่ใช้ในการเก็บตัวอย่างตรวจจนถึงทราบผลการเพาะเชื้ออยู่ที่ 4 วัน (IQR 3.0–5.0) ส่งผลให้ผู้ป่วยได้รับการรักษาล่าช้า ในจำนวนผู้ป่วย 876 รายนี้ พบว่ามีผู้ป่วย 199 ราย (23%) เสียชีวิตก่อนได้รับการยืนยันผลการเพาะเชื้อ ส่วนผู้ป่วยจำนวน 433 รายที่เข้าร่วมโครงการ พบว่าผู้ป่วยจำนวน 114 ราย (26%) เสียชีวิตภายใน 28 วันแม้จะได้รับการรักษาหลังทราบผลการเพาะเชื้อ คณะผู้วิจัยจึงได้พัฒนาชุดตรวจ CRISPR-BP34 เพื่อช่วยย่นระยะเวลาในการวินิจฉัยและให้การรักษาโรคได้รวดเร็วขึ้น โดยคณะผู้วิจัยได้ทำการเก็บสิ่งส่งตรวจจากผู้ป่วยโรคเมลิออยด์จำนวน 114 ราย และจากผู้ป่วยโรคอื่นอีกจำนวน 216 ราย (รวม 330 ราย) ระหว่างวันที่ 26 พฤษภาคม พ.ศ. 2565 ถึง 31 ธันวาคม พ.ศ. 2565 และทำการทดสอบประสิทธิภาพของชุดตรวจ CRISPR-BP34 เทียบกับวิธีการเพาะเชื้อ ผลการทดสอบพบว่าชุดตรวจ CRISPR-BP34 สามารถลดระยะเวลาที่ใช้ในการวินิจฉัยโรคเมลิออยด์เหลือ 1.1 วัน (IQR 0.7–1.5) เมื่อทำการทดสอบกับตัวอย่างเลือด ใช้เวลา 2.3 ชั่วโมง (IQR 2.3–2.4) เมื่อทดสอบกับตัวอย่างปัสสาวะ และ 3.3 ชั่วโมง (IQR 3.1–3.4) เมื่อทดสอบกับตัวอย่างสารคัดหลั่งจากระบบทางเดินหายใจ น้ำหนอง และสารคัดหลั่งอื่นๆ เมื่อวิเคราะห์ผลการทดสอบโดยรวม พบว่าชุดตรวจ CRISPR-BP34 มีความไวอยู่ที่ 93.0% (106 จาก 114 ตัวอย่าง [95% CI 86.6–96.9]) เทียบกับความไวของวิธีเพาะเชื้อที่

66.7% (76 จาก 114 ตัวอย่าง [57.2–75.2]) และชุดตรวจ CRISPR-BP34 มีความจำเพาะอยู่ที่ 96.8% (209 จาก 216 ตัวอย่าง [95% CI 93.4–98.7]) เมื่อเทียบกับวิธีการเพาะเชื้อที่มีความจำเพาะ 100% (216 จาก 216 ตัวอย่าง [98.3–100.0])

สรุปผล

จากการศึกษานี้พบว่าชุดตรวจ CRISPR-BP34 มีความไว ความจำเพาะ และความรวดเร็วในการวินิจฉัยโรคเมลิออยด์ดีกว่าการเพาะเชื้อ ดังนั้นชุดตรวจ CRISPR-BP34 นี้จึงมีศักยภาพที่จะพัฒนาเป็นชุดตรวจวินิจฉัยโรคเมลิออยด์ ณ จุดคัดกรองคนไข้ได้ ในขั้นตอนต่อไป คณะผู้วิจัยจะมุ่งเน้นในเรื่องของการขยายฐานการผลิตและการลดต้นทุน เพื่อให้ชุดตรวจมีราคาที่ถูกลงและผู้ป่วยสามารถเข้าถึงได้

ผู้สนับสนุนทุนวิจัย

มหาวิทยาลัยเชียงใหม่ และเวลล์คัมทรัสต์ สหราชอาณาจักร (Wellcome Trust, UK)
