# Supplementary material for: Benchmarking CRISPR-BP34 for point-of-care melioidosis detection in low-income and middle-income countries: a molecular diagnostics study
Source: Lancet Microbe. 2024 Apr;5(4):e379–89. doi: 10.1016/S2666-5247(23)00378-6 (PMC10990966; doi:10.1016/S2666-5247(23)00378-6)
Supplement: Supplementary appendix 2 [file mmc2.pdf]

THE LANCET
Microbe

Supplementary appendix 2

This appendix formed part of the original submission and has been peer reviewed.
We post it as supplied by the authors.

Supplement to: Pakdeerat S, Boonklang P, Angchagun K, et al. Benchmarking CRISPR-BP34 for point-of-care melioidosis detection in low-income and middle-income countries: a molecular diagnostics study. *Lancet Microbe* 2024. https://doi.org/10.1016/S2666-5247(23)00378-6

SUPPLEMENTARY APPENDIX

Supplement to

Benchmarking CRISPR-BP34 for point-of-care melioidosis detection in LMIC: a molecular diagnostics study

Sukripong Pakdeerat Bsc; Phumrapee Boonklang MPhil; Kesorn Angchagun Bsn; Chalita Chomkatekaew Msc; Navaporn Apichaidejudom Bsc, Yaowaret Dokket Bsc, Areeya Faosap Bsc, Gumphol Wongsuwan Msc; Vanaporn Wuthiekanun Bsc, Panatda Aramrueung Msc; Phadungkiat Khamnoi Bsc; Hathairat Thananchai DPhil; Suwattiya Siriboon MD; Parinya Chamnan MD, PhD; Sharon J Peacock MD, PhD; Nicholas PJ Day MD; Nicholas R Thomson PhD; Chayasith Uttamapinant PhD; Somsakul Pop Wongpalee PhD*; Claire Chewapreecha PhD*

*Indicates equal contribution and corresponding authors: somsakul.w@cmu.ac.th or pop.wongpalee@gmail, and claire@tropmedres.ac

TABLE OF CONTENT

SUPPLEMENTARY METHODS 4

Study 1 Protocol: a prospective observational cohort study of patients with culture-confirmed melioidosis 4

Study design, site, and population 4

Participant inclusion criteria 4

Participant exclusion criteria 4

Screening 4

Ethics/protection of human subjects 4

Observed symptoms and organ involvement of melioidosis patients 5

Categorising clinical spectrum of melioidosis 5

Culture-confirmed assay used to screen patients 6

Antibiotics prescribed, dose, and duration of melioidosis treatment following the arrival of culture-confirmed diagnosis 6

Study 2 Protocol: a diagnostic evaluation of the sensitivity and specificity of CRISPR-BP34 assay 8

Production of CRISPR-Cas12a 8

CRISPR RNA synthesis 8

Generation of genetically-modified *E. coli* strain 8

Rationale for using *E. coli* as a surrogate for *B. pseudomallei* 9

CRISPR-BP34 assay 9

qPCR validation 11

Suggestions to mitigate DNA cross-contamination 11

Assessment of CRISPR-BP34 by WHO's ASSURED guideline and strategies to drive it to near-point of care test 12

SUPPLEMENTARY FIGURES 13

Supplementary Figure S1. A setup of a workstation for CRISPR-BP34 assay 13

Supplementary Figure S2. A workflow of CRISPR-BP34 assay 14

Supplementary Figure S3. CRISPR-BP34 assay flowchart for hemoculture samples or blood 15

Supplementary Figure S4. CRISPR-BP34 assay flowchart for genitourinary samples 16

Supplementary Figure S5. CRISPR-BP34 assay flowchart for bodily fluids (such as sputum or pus) 16

Supplementary Figure S6. RPA reaction assay flowchart 18

Supplementary Figure S7. CRISPR reaction and dipstick detection assay flowchart 19

Supplementary Figure S8. Kaplan-Meier curves of demographic status and antibiotics prescription associated with melioidosis mortality post admission date 20

Supplementary Figure S9. Time taken for the culture and the CRISPR-BP34 approaches, and case examples 20

Supplementary Figure S10. Comparison of qPCR cycle threshold (ct scores) between culture positive and negative samples 22

Supplementary Figure S11. Symptoms and types of melioidosis reported in the cohort. 23

Supplementary Figure S12. Other pathogens reported in non-melioidosis controls 24

SUPPLEMENTARY TABLES 25

Supplementary Table S1. A list of primers and oligos used in the study 25

Supplementary Table S2 Cox regression analysis evaluating demographic status and antibiotics prescribed on first day of admission associated with mortality 27

Supplementary Table S3. Clinical sensitivity and specificity of culture and CRISPR-based methods......... 29

Supplementary Table S4. Stages of CRISPR-BP34 development by WHO ASSURED guideline 30

SUPPLEMENTARY METHOS

Study 1 Protocol: a prospective observational cohort study of patients with culture-confirmed melioidosis

Study design, site, and population

The objective of the study was to examine the clinical and genetic factors that influence disease outcomes (28-day mortality) in melioidosis. To achieve this, the study gathered clinical data including the time taken to reach melioidosis diagnosis, medical history, symptoms, and treatments received; bacterial samples, individual blood samples from both melioidosis cases and control cases; and 28-days mortality outcomes. To mitigate follow-up loss bias, participants were kept informed and contact information was updated. The patients involved in this study were recruited from Sunpasitthiprasong Hospital in Ubon Ratchathani, where high incidence of melioidosis is observed. Additionally, the hospital houses the satellite unit of the Mahidol Oxford Tropical Medicine Research Unit (MORU) that facilitated the processing of clinical samples. Melioidosis patients were recruited from the Hospital Infectious department. The recruitment phase of the study lasted for three years (October 2019 - December 2022), covering three monsoon seasons when a rise in melioidosis cases was anticipated. The full study protocol is available in Angchagun *et al.* 2023[1]. Only the data concerning the host characteristics of the melioidosis patients was described in this paper.

Participant inclusion criteria

-Age ≥ 18 years
-Culture-confirmed infection of *Burkholderia pseudomallei* from any clinical samples.
-Willingness to participate in the study and written, informed consent obtained from the patient or their relative.
-Resident in Northeast Thailand for at least the previous 2 years

Participant exclusion criteria

-Current TB or treatment of TB within the last 6 months
-Documented HIV infection, chemotherapy, or other immunosuppressant therapy in the last 12 months.
-Pregnancy
-Current covid19 (due to the recruitment during covid pandemic)*

Screening

All patients at Sunpasitthiprasong Hospital with a culture-confirmed diagnosis of suspected melioidosis were approached by a member of study staff. The patient were screened for eligibility. A written participant information sheet (PIS) and informed consent form (ICF) written in Thai language were presented to the participant or relative where they lack capacity detailing no less than: the exact nature of the study; the implications and constraints of the protocol; the known side effects and any risks involved in taking part. The participant or relative were allowed adequate time (minimum of ten minutes) to consider the information, and the opportunity to question the Investigator or other independent parties to decide whether they will participate in the study. Written informed consent were then be obtained by means of participant or relative dated signature and dated signature of the person who presented and obtained the informed consent. If the patient or relative is illiterate, a thumbprint was obtained. A copy of the signed Informed Consents was given to the participant or responsible relative. The original signed forms has been retained at the study site.

Ethics/protection of human subjects

The study received ethical approval from the Sunpasitthiprasong Hospital Ethical Review Board (015/62C) and the Oxford Tropical Research Ethics Committee (OxTREC 25-19). All research data sets arising from the study were pseudonymised. This allowed for eventualities such as feedback of individual data or withdrawal of consent. However, subject names or identities of participants will not be revealed.

Observed symptoms and organ involvement of melioidosis patients

Melioidosis patients exhibit a wide range of clinical symptoms. Individuals may experience a combination of more than one of the following symptoms[2]. The frequency of each and combination of symptoms are reported in **Supplementary Figure S11**.

i. High and persistent fever: Fever is the most common symptom with melioidosis fever often lasting several days or weeks. The fever may start abruptly with chills and sweating. In melioidosis endemic areas such as our setting in Ubon Ratchathani, it can be challenging to discriminate melioidosis from other febrile illnesses based on fever alone.

ii. Respiratory symptoms: the severity of the symptom can vary depending on the location of infection and the bacterial loads. The common symptoms included persistent cough, chest pain, shortness of breath, hemoptysis or pneumonia.

iii. Skin lesions: the severity of the symptom can vary depending on the location. This can be manifested as small localised abscesses to large necrotic ulcers. Skin lesions can present with swollen areas on the skin and can be accompanied by lymphadenopathy.

iv. Gastrointestinal symptoms: the severity can vary depending on the extent of infection. The symptoms typically include abdominal pain, nausea and vomiting, diarrhea (can be watery or bloody), anorexia (which results in weight loss), gastrointestinal bleeding (which leads to anemia) and hepatomegaly.

v. Muscle and joint pain: Joint pain could either be localised to one area or affect multiple joints. The symptoms can be accompanied by stiffness, swelling and redness in the affected area. Similarly, muscle pain can be localised or generalised over the entire body, and can be accompanied by muscle weakness and fatigue.

vi. Neurological symptoms (reported in Australia with a small number of cases documented in Thailand): this included headache, seizures, altered mental status, cranial nerve dysfunction, motor weakness, sensory disturbances, and ataxia.

Categorising clinical spectrum of melioidosis

The clinical spectrum of melioidosis observed in our cohort can also be categorised into four distinct yet interconnected groups as the following:

Pulmonary Infection (52%): This category is marked by symptoms such as cough, chest pain during breathing, high fever, and it is typically acquired through inhalation or hematogenous spread post-inoculation.

Bloodstream Infection (69%): Confirmed by positive blood culture during admission, this category is characterised by fever with shivers and sweating, upper abdominal pain, diarrhea, joint pain, muscle tenderness, and disorientation.

Disseminated Infection (33%): In this group, patients exhibited fever, weight loss, abdominal pain, muscle and joint pain, headaches, along with clinical signs of abscess formation in multiple organs, with or without bacteremia.

Localised Infection (22%): Cases in this category manifested as skin ulcers, subcutaneous tissue abscesses, and parotid abscesses.

This classification provides a comprehensive understanding of the diverse ways melioidosis presents itself in patients, which is also consistent with previous reports from Thailand and neighbouring countries[3,4].

Culture-confirmed assay used to screen patients

We collected clinical specimens from patients suspected to have meloidosis including blood, urine, respiratory secretion and bodily fluid samples using WHO's guideline[5]. These specimens were processed for culture-confirmed diagnosis as following:

Blood sample

As the amount of *B. pseudomallei* cells directly recovered from patient blood is low (1 CFU/mL), an enrichment process is required. Peripheral blood samples were aseptically drawn from venipuncture sites and transferred into BACTEC^{TM} Plus Aerobic/F Culture Vials catalogue number 442192 (Becton Dickinson, Cockeysville, MD, USA). Blood culture bottles were incubated at 35 °C in the BACTEC 9120 instrument until a positive result or for a maximum of 5 days. As part of the laboratory routine, the causative pathogens were identified from positive bottles by subculturing the positive specimens on blood agar plates. The plates were incubated at 37 °C and inspected daily for the appearance of colonies, typically within 1-2 days (**Figure 3A**).

Urine sample

A urine sample was collected from a patient suspected of having melioidosis. As a part of routine practices, 1 µgL of an unprocessed urine sample (neat sample) was directly spread onto a MacConkey and an Ashdown's agar plates to test for the presence of *B.pseudomallei* and to quantify bacterial load per sample. Urine sample was centrifuged at 3,000 g for 5 minutes. 10 µL of urine pellet was streaked to an Ashdown's agar plate. Both plates were incubated at 37 °C for 2 (MacConkey agar) and 4 days (Ashdown's agar), respectively. The culture was inspected daily for colonies of *B. pseudomallei*.

Respiratory secretion and bodily fluid sample

Respiratory secretion samples (sputum, tracheal suction and pleural fluid) and bodily fluid samples (pus, tissue specimen, synovial fluid or other fluid) were collected from patients suspected of having melioidosis. Using a sterile loop, a neat sample (10 µL) was plated on blood agar, MacConkey agar and Ashdown's agar plates to test for the presence of *B. pseudomallei* as well as to quantify bacterial load per sample. A neat sample was also inoculated into selective broth. Both the plates and the broth were incubated at 37 °C for 2 days and inspected daily for positive culture. The positive broth culture was subsequently subcultured onto Ashdown's agar plate to identify bacterial species.

Antibiotics prescribed, dose, and duration of melioidosis treatment following the arrival of culture-confirmed diagnosis

Upon receiving culture-confirmed results of melioidosis, Sunpasittihiprasong Hospital strictly follows the treatment protocol outlined by the Thailand Ministry of Public Health[6]. The treatment for adults can be divided into the initial intensive phase of treatment with intravenous ceftazidime for 10 to 14 days to mitigate sepsis-related risks, and eradication phase with oral co-trimoxazole for 12 to 20 weeks to prevent melioidosis relapse.

<u>The initial intensive phase with intravenous treatment</u>

This regimen spans a duration of 10 to 14 days, patients are administered primarily with ceftazidime intravenously at a dose of 100-120 mg/kg/day every 8 hours at a duration of 10 to 14 days. If ceftazidime is ineffective, alternative antibiotics can be prescribed as follow:

-Imipenem at 50 mg/kg/day every 8 hours.

-Meropenem at 75 mg/kg/day every 8 hours

-Cefoperazone-sulbactam at 25 mg/kg/day every 8 hours. However, this is not suitable for central nervous system melioidosis due to limited spinal fluid diffusion.

-Amoxicillin-clavulanate (co-amoxiclav) at amoxicillin/clavulanate 120/30 mg/kg/day every 4 hours. This is reserved as last choice due to higher treatment failure and resistance. Also, this drug is not recommended for neurological symptoms due to poorer rate of drug diffusion into the spinal fluid.

The eradication phase with oral treatment

Given the potential relapse in 3-29% of patients, a treatment duration of 12-20 weeks is crucial to ensure effective bacterial clearance. During this phase, patients receive primary oral treatment with Co-trimoxazole (sulfamethoxazole-trimethoprim) at a dose of 8 – 10 mg/kg/day (80 mg trimethoprim/400 mg sulfamethoxazole). In cases where resistance to Co-trimoxazole is encountered, alternative drugs can be prescribed with compromised success rates:

-Co-amoxiclav: Amoxicillin 60 mg/kg/day and clavulanate 15 mg/kg/day.

-Ciprofloxacin 500 mg twice a day. This option is reserved as the last resort when Co-trimoxazole and Co-amoxiclav prove ineffective.

The choice between the 12 or 20-week treatment duration depends on the extent of organ involvement. Patients with neurological, testis, bone infections, or mycotic aneurysms should complete the oral treatment for a full 20 weeks to ensure complete bacterial clearance.

Study 2 Protocol: a diagnostic evaluation of the sensitivity and specificity of CRISPR-BP34 assay

To improve the melioidosis diagnosis observed in study 1, an alternative CRISPR-BP34 assay was performed on frozen specimens obtained from patients suspected of having melioidosis in the same hospital setting. The results and time taken to process the samples were recorded to enable the head-to-head comparison of the CRISPR-BP34 and the routine culture performance. The study was granted permission to use clinical samples as well as ethical approval from the Ethical Review Board of Sunpasitthiprasong Hospital (029/65C). The study also received ethical exemption from Chiang Mai University (9190/2565) for secondary use of biospecimens. The laboratory protocol was described as below.

Production of CRISPR-Cas12a

Expression and purification of MBP-LbCas12a

The methods for expression and purification followed the same procedures as described in the previous study[7].

CRISPR RNA synthesis

The CRISPR RNA, crBP34, was reported previously[7] and used in this study. The method for crRNA synthesis followed the same procedures as described in the previous study.

Generation of genetically-modified *E. coli* strain

This genetically modified *E. coli* strain was used for spiking in blood and urine samples to determine molecular sensitivity of the CRISPR-Cas12a detection procedure. This strain was created using λ Red recombinase-mediated DNA recombination[8] at the *lacZ* locus in a parental *E. coli* strain, NEB 10-beta (NEB, USA, #C3019H). The target RPA amplicon of the *B. pseudomallei* was first amplified by PCR using KOD-Plus-Neo (Toyobo, Japan, #KOD-401) and primers containing adapter sequences (primer 200/201). The PCR amplicon was gel purified by TIANgel Midi Purification kit (TIANGEN, China, #4992443) and cloned into pACYCDuet-1 (Novagen, USA, #71147) at NcoI restriction site (ThermoFisher Scientific, USA, #ER0571). The DNA sequences in the plasmid that encompassed the RPA amplicon and Chloramphenicol resistance gene were then PCR amplified from the plasmid (using primer 221/222) and subsequently gel purified. This PCR amplicon represents the DNA that will be inserted into the lacZ locus using λ Red recombinase-mediated DNA recombination. This process requires homology arms on both the upstream and downstream of the insertion point. To accomplish this, ~500-bp DNA sequences of the upstream and downstream regions were separately amplified from the genomic DNA of the *E. coli* NEB 10-beta strain using PCR. The two resulting PCR amplicons were then gel purified. It is important to note that some of these primers that were used in these PCR reactions (primer 217/218 and 219/220) contained overlapping sequences with the DNA was intended to be inserted, allowing the three PCR amplicons to be assembled into a targeting cassette in a subsequence PCR reaction (using primer 217/220)[9].

To insert the targeting cassette into the *lacZ* locus, a modified protocol derived from previous studies was developed[8,10]. Briefly, pKD46 (a gift from Dr. Aiyada Aroonsri, BIOTEC, Thailand), which expresses λ Red recombinase enzymes, was electrotransformed into NEB 10-beta *E. coli* using 1.8 kV, 200 Ω, 25 μF parameters. The cells were grown overnight on LB agar (Miller) (Titan, India, #TM376) in the presence of 100 μg/mL Ampicillin (AppliChem, Germany, #A0839) at 30 °C, and a single colony was selected and grew a starter culture. This starter culture was inoculated into 150-mL LB broth (Miller) (Titan, India, #TM406) in the presence of Ampicillin and 10 mM L-arabinose (TCI, Japan, #A0515) to induce expression of the λ Red recombinase enzymes. Cells were allowed to grow at 30 °C until $O.D._{600}$ reached 0.6. They were then harvested and washed three times in an ice-cold solution of 10 %v/v glycerol to ensure complete removal of any impurities, and finally they were resuspended in 1 mL of the same solution. 1.0-1.5 μg of the purified targeting cassette was electroporated into 40 μL of these cells. The cells were recovered in LB (Miller) broth at 37°C for 3 hours with gentle agitation and then plated on LB (Miller) agar containing 5 μg/mL Chloramphenicol (AppliChem, Germany, #A1806) overnight at 37°C. All visible colonies were picked and re-streaked onto LB (Miller) agar containing 7 μg/mL Chloramphenicol to remove any contaminant cells. The genomic DNA of the genetically modified *E. coli* was extracted using the GeneJET

Genomic DNA Purification Kit (ThermoFisher Scientific, USA, #K0721). The DNA was then subjected to PCR (using primer 198/199), and the amplicons were gel purified and sent for sequencing to confirm the accuracy of the inserted DNA sequence. Correct clones were stored in 20 %v/v glycerol at -80°C.

Rationale for using *E. coli* as a surrogate for *B. pseudomallei*

For safety reasons, we used *E. coli* as a substitute to assess the assay's limit of detection in the spiking experiment. Although no prior study directly compared DNA extraction yields between *E. coli* and *B. pseudomallei*. Kobayashi *et al*[11] found comparable DNA recovery from genetically modified *E. coli* and their target Gram-negative bacterium, *Bacteroides fragilis* of similar genome size. This discovery, coupled with the shared cellular structure among Gram-negative bacteria, implies that *E. coli* could serve as a useful control for DNA extraction in Gram-negative bacterial species. However, the disparity in genome size between *E. coli* (4.5 to 5.5 Mbp) and *B. pseudomallei* (7.3 Mbp) might result in non-specific binding of the RPA primers to the DNA of *B. pseudomallei*, leading to the reduction of RPA amplification and assay efficiency. Yet, when considering the substantial size and prevalence of host cells' DNA that could potentially contaminate the same clinical samples (3,100 Mbp and in greater abundance), the difference in genome size of bacterial species carrying the DNA might prove negligible. In light of these considerations, our decision was to employ *E. coli* as a proxy for *B. pseudomallei*, enabling us to explore the detection threshold of the CRISPR-BP34 assay within this specific spiking experiment.

CRISPR-BP34 assay

Sample preparation

For urine sample, 5 mL of urine sample was centrifuged at 3,000 g for 5 minutes. The supernatant was carefully removed, and the sample pellet was resuspended in 1 mL of PBS The resuspended sample was then transferred to a new microcentrifuge tube and centrifuged at 10,000 g for 5 minutes. The supernatant was again carefully removed, leaving approximately 20 µL on top of the sample pellet. This sample was then used for the CRISPR-BP34 assay, as outlined in the protocol provided below and in **Supplementary Figure S4**. For blood culture, respiratory secretion samples and other body fluid, a 200-µL aliquot from samples can be used directly. If the sample was viscous, 1 mL of PBS was used to dilute the sample before transferring. This sample was then proceeded to CRISPR-BP34 assay outlined below and in (**Supplementary Figure S5**)

After sample preparation, 200 µL of the clinical sample was aliquoted into a 1.5 mL test tube. We used 200 µL sample volume because of convenience in sample handling; however, a larger volume is also possible to perform this step. This CRISPR-BP34 assay consists of (i) host cell depletion, (ii) DNA extraction, (iii) RPA and (iv) CRISPR reaction and detection

Host cell depletion

This step is required for samples that are contaminated with significant amounts of human host cells, for example, blood, hemoculture, sputum and pus. Contamination of host cells' genomic DNA negatively impacts sensitivity and specificity of nucleic acid amplification[12–16]. Since bacterial pathogens contain a rigid cell wall, depletion of host cells' DNA can be simply achieved by selectively lysing the mammalian cells with a mild detergent-containing buffer followed by centrifugation to collect intact bacterial cells before proceeding to DNA extraction. To deplete host cells, an equal volume of 200 µL of a Mammalian Cell Lysis Buffer-1[17] (MCLB-1, containing 2 M Na_2CO_3 pH 9.8 and 1% v/v Triton-X 100) was added to the test tube. The test tube was then vigorously vortexed every 15-20 seconds during a 4-minute incubation at room temperature. The mixture was neutralised with a Neutralizing buffer (Tris-HCl pH 4.5), briefly vortexed and centrifuged at 10,000 g for 5 minutes. The supernatant containing DNA and cytoplasmic contents of human cells was carefully discarded without disturbing a bacteria-containing pellet at the bottom. This pellet was then washed with 1 mL PBS, briefly vortexed and centrifuged at 10,000 g for 5 min. This washing step could be repeated one more time if the wash supernatant was still contaminated with host cells' constituents such as blood

pigments. The wash supernatant was carefully removed and left behind ~20 µL atop the bacteria-containing pellet. This host cell-depleted sample was then proceeded to the DNA extraction step. Technical details of the host cell depletion step were presented in **Supplementary Figure S3** and **S5**.

DNA extraction from clinical samples

While a simple and streamlined methodology for extracting pathogen DNA is preferred, some clinical samples remain complex in nature even after the host cell depletion step. These samples prove difficult to be processed by the simple extraction method. In our protocol, bacterial DNA can be easily extracted by alkaline lysis if the bacteria-containing pellet is less than 10 µL in volume. This method has been successfully applied for urine, whole blood, and hemoculture samples. However, samples such as sputum, pus, and respiratory secretions remain viscous and large in volume even after host cell depletion, necessitating a more thorough extraction method using a commercial spin column. To facilitate sample processing, we have developed pipelines that guide the processing of different clinical samples using easy-to-follow flowcharts, which can be found in **Supplementary Figure S3, S4** and **S5**.

Alkaline lysis

This DNA extraction method is a modified version of a method derived from a patent[18], and was used to extract DNA from a bacteria-containing pellet that was less than 10 µL in size. Typically, these pellets were obtained from centrifuged urine samples or host-cell depleted whole blood or hemoculture samples. To perform this step, 2 µL of 550 mM NaOH (i.e. alkaline) was added and mixed with the pellet. This resulted in a final concentration of 50 mM NaOH. The sample was heated at 70 °C for 10 minutes to lyse bacterial cells, after which 2.5 µL of 1 M Tris pH 7.5 was added to neutralize the sample (final Tris concentration ~100 mM). The resulting sample could be used directly in a RPA reaction or stored at -20 °C until needed. Technical details of this step were presented in **Supplementary Figure S3** and **S4**.

GeneJet spin column

This extraction method was utilised for extracting DNA from bacterial-containing pellets larger than 10 µL. Such pellets could be derived from centrifuged urine samples, host-cell depleted whole blood, or hemoculture samples. Bodily fluids such as pus, sputum, and respiratory secretions always yield a bacterial-containing pellet larger than 10 µL after the host cell depletion step; therefore, they are always processed using this extraction method. To purify the genomic DNA, the GeneJet Genomic DNA Purification Kit (ThermoFisher Scientific, USA, #K0721) was used with a modified protocol as follows. The pellet was resuspended in 200 µL of PBS, and 200 µL of lysis buffer along with 20 µL of proteinase K were added. The mixture was incubated at 56 °C for 30 minutes. Then, 20 µL of RNase A was added, and the mixture was further incubated at 37 °C for 10 minutes. Next, 400 µL of 50 %v/v ethanol was mixed in, and the resulting mixture was transferred to the spin column and washed following the manufacturer's protocol. The DNA was eluted in only 30 µL of an elution buffer at 56 °C for 2 minutes, before being spun down to a new test tube at 12,000 g for 1 minute. The DNA was stored at -20 °C until use. Technical details of this step were presented in **Supplementary Figure S5**.

Recombinase polymerase amplification (RPA)

TwistAmp Basic (TwistDx, USA, #TABAS03KIT) was used for RPA in accordance with the manufacturer's protocols, with modifications as follows. (i) The total volume of an RPA reaction was adjusted to 30 µL, (ii) incubation was performed at 39℃ for 30 minutes, (iii) DNA input was only 2 µL (adding more DNA to the reaction did not necessarily increase sensitivity, as it could contain some inhibitors) and (iv) MgOAc was added last to initiate the reaction. All RPA reactions were stored at -20℃ until used. Technical details of this step were presented in **Supplementary Figure S6**. RPA primers, 148 and 149, were previously reported[7], synthesised and purified by standard desalting by Macrogen (Korea).

CRISPR reaction and dipstick detection

CRISPR reactions were performed in 50-µL volume that contained 100 nM crRNA, 200 nM MBP-LbCas12a, 100 nM FAM-biotin probe, 5 µL RPA reaction and 1x HOLMES buffer 1[19] (2 mM spermidine, 40 mM Tris pH 8.5, 6 mM

$MgCl_2$, 1 mM DTT, 40 mM glycine, 0.001 %v/v Triton X-100, 0.4 %w/v PEG-20,000). The reaction was incubated at 37 °C for 60 minutes. Thereafter, a lateral flow dipstick (Milenia Biotec, Germany, #MGHD1) was directly dipped into the reaction and allowed to develop for 5 minutes before reading. Technical details of this step were presented in **Supplementary Figure S7**.

Determining the bacterial loads from clinical specimens

The number of colony forming units (CFU/mL) of *B. pseudomallei* was estimated from the positive agar plates from hemoculture; genitourinary fluid; respiratory secretion; and pus, tissue and other body fluid samples as part of the laboratory practice. The number of bacterial cells in direct blood samples was aggregated from the former dataset[20]. Blood collected directly from patients who had not been treated and suspected to have melioidosis was transferred into isolator tubes. The blood was then lysed to release intracellular bacterium, and centrifuged to condense the number of bacterial cells before being plated on blood agar plates. The CFU was counted.

qPCR validation

Three sets of qPCR primers, namely 192/193, 270/271, and 274/275, were utilised to detect *B. pseudomallei* genomic DNA in samples that produced inconsistent results between CRISPR-BP34 assay and either standard culture-confirmed assay or hemoculture. These samples were usually positive for the CRISPR assay but negative for the culture methods, likely due to their low sensitivity. The three primer pairs target two different genes in order to increase the likelihood of detection, as a meta analysis indicated that targeting a single gene TTS1 resulted in a qPCR sensitivity of only 77.1%.[21] These three primer pairs are listed in **Supplementary Table S1** and were synthesised and desalted by Macrogen (South Korea). The qPCR reactions were prepared in a 20-µL volume using Maxima SYBR Green/ROX qPCR Master Mix (ThermoFisher Scientific, USA, #K0221), following the manufacturer's instructions. Duplicated qPCR reactions were carried out on the Applied Biosystems 7500 Fast Real-Time PCR System using the following conditions: an initial denaturation step of 95 °C for 10 minutes, followed by 40 cycles of denaturation at 95 °C for 15 seconds, annealing at 61 °C for 30 seconds (for primer pairs 192/193) or 64°C (for primer pairs 270/271) or 62 °C (for primer pairs 274/275), and extension at 72 °C for 30 seconds. Positive detection was determined if any duplicate reaction in any primer pairs yielded a positive result. Standard melting curve analysis was performed at the end of the PCR and compared to that of positive control reactions. ΔRn threshold was automatically calculated by the built-in program.

Suggestions to mitigate DNA cross-contamination

Like other nucleic acid amplification assays, such as PCR and LAMP, RPA is also susceptible to the problem of false positives caused by amplicon cross-contamination. To minimize this risk during the RPA step, we suggest implementing the following practices.

i. Assign a dedicated workstation for the RPA step, if possible, and clean the workstation before and after the RPA step
ii. Avoid air turbulence, such as in a biosafety cabinet, when performing this step or when opening an RPA test tube
iii. Use filter pipette tips and have a dedicated disposal bin for pipette tips that have come into contact with the RPA reaction
iv. Even though we did not perform this, RPA and CRISPR reactions can be coupled into a single 'one-pot' reaction[16]. This eliminates the need to open the RPA test tube, but a fluorescent probe and compatible readout must be utilised
v. It is also possible to routinely incorporate deoxyuridine triphosphate (dUTP) during the RPA step and treat DNA samples with Uracil DNA Glycosylase (UDG) before the RPA reaction[17]. UDG cleaves uracil-containing amplicons, thus reducing cross-contamination.

Assessment of CRISPR-BP34 by WHO's ASSURED guideline and strategies to drive it to near-point of care test

The World Health Organisation (WHO) outlines the key features of an effective point-of-care (POC) diagnostic test for low- and middle-income countries using specific criteria: Affordability (A), Sensitivity (S), Specificity (S), User-friendly (U), Rapid and robust (R), Equipment-free (E), and Deliverable (D), also known as ASSURED. To further improve diagnostic capabilities, Land *et al.* 2019[22] introduced two more important criteria, referred to as "REASSURED": Real-time connectivity (R) and Ease of specimen collection and processing (E). **Table S3** provides an overview of how CRISPR-BP34 aligns with each of these REASSURED criteria.

In terms of technological improvements, although the current CRISPR-BP34 test does not fully meet all criteria, we are making it very close to a near POC test. Our plans include the following:

1. Swtiching from liquid reagents to a freeze-dried form, making them instantly usable by adding water and DNA, thereby eliminating various pipetting steps and the need for cold storage

2. Combining RPA and CRISPR reaction into a single step (one-pot reaction) to reduce assay time and increase ease of operation. One drawback, however, is that the sensitivity may be negatively impacted by a compromised one pot condition[23].

3. Partnership with industry to mass-produce and lower the costs, aiming for less than 10 US dollars per test. This will make CRISPR-BP34 accessible for various settings, from district healthcare labs to primary care facilities.

We are also working with the Thai Ministry of Public Health to advocate for integrating CRISPR-BP34 alternatives in pilot hospitals with high melioidosis cases. This collaboration will help us collect user feedback, refines the prototype, and trains personnel in CRISPR techniques. This strategy ensures the successful implementation of the CRISPR-BP34 test in the real clinical settings.

SUPPLEMENTARY FIGURES

(for greater resolution of Figure S1 – S7, please refer to https://doi.org/10.6084/m9.figshare.23997999)

Supplementary Figure S1. A setup of a workstation for CRISPR-BP34 assay

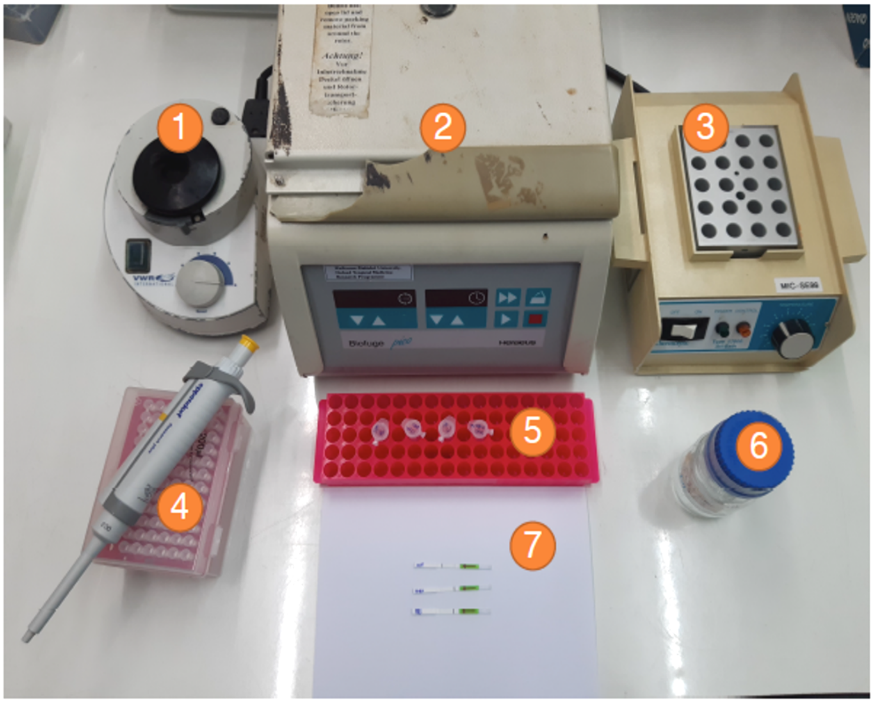

1) Vortex
2) Centrifuge
3) Heat block
4) Pipette and tips
5,6) Reagents
7) Lateral flow dipsticks

+ Waste bin dedicated for RPA waste is recommended

Supplementary Figure S2. A workflow of CRISPR-BP34 assay

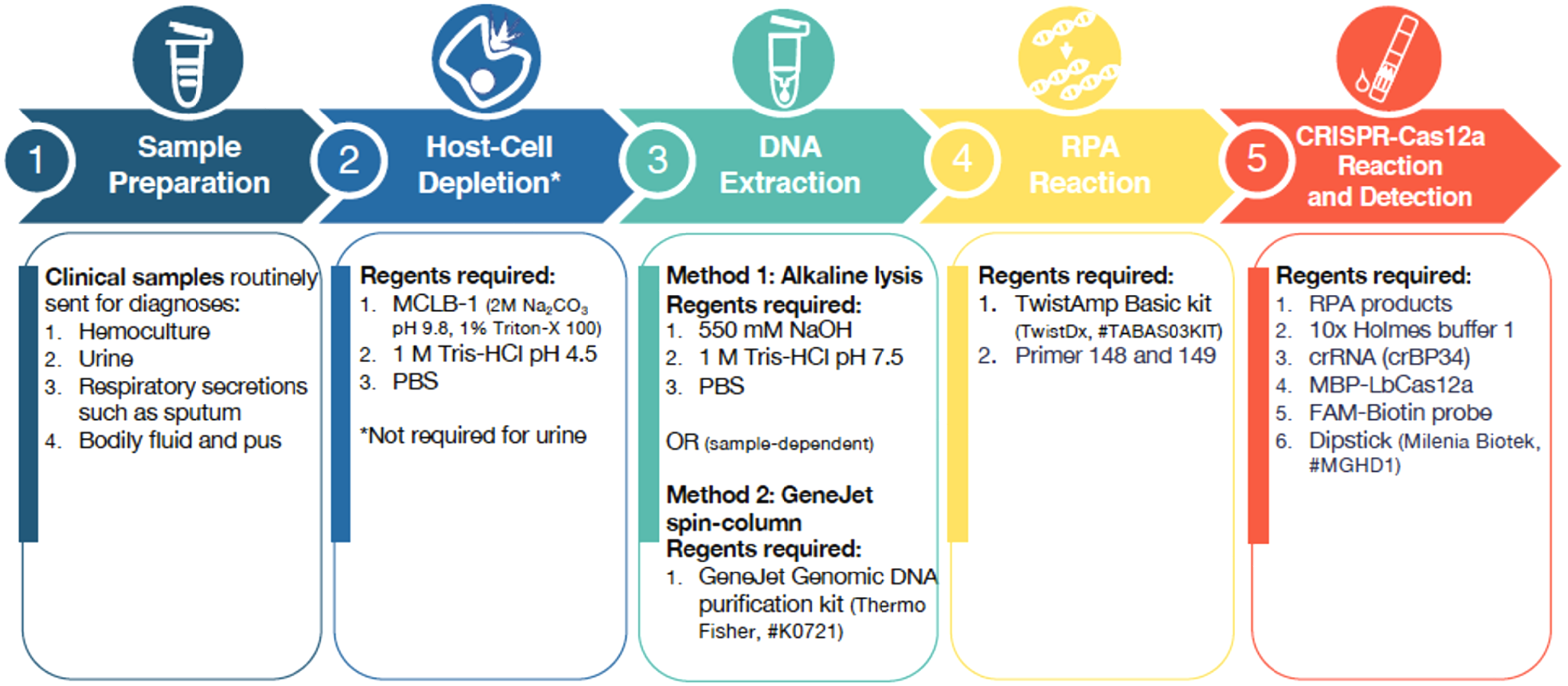

Supplementary Figure S3. CRISPR-BP34 assay flowchart for hemoculture samples or blood

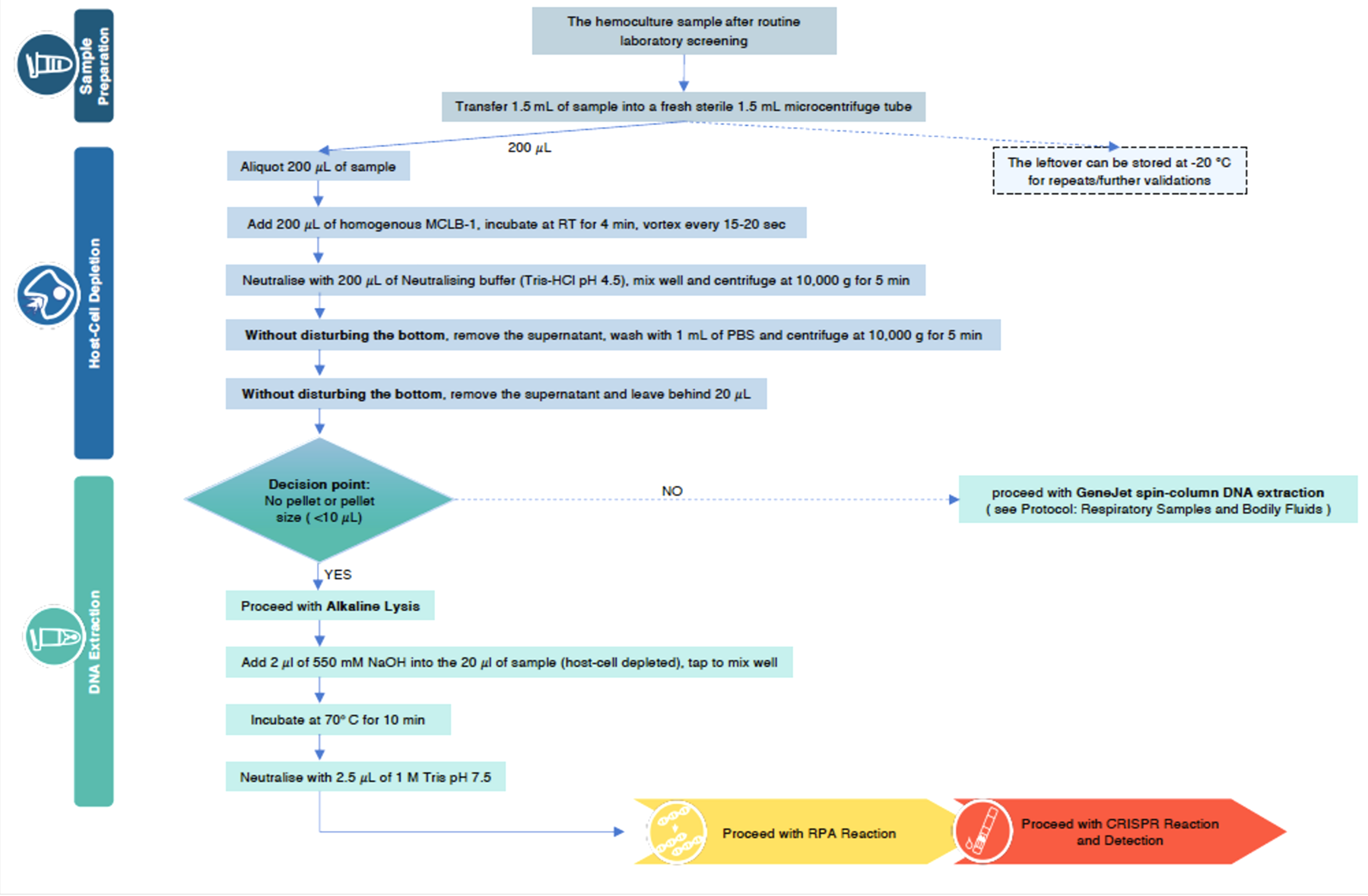

Supplementary Figure S4. CRISPR-BP34 assay flowchart for genitourinary samples

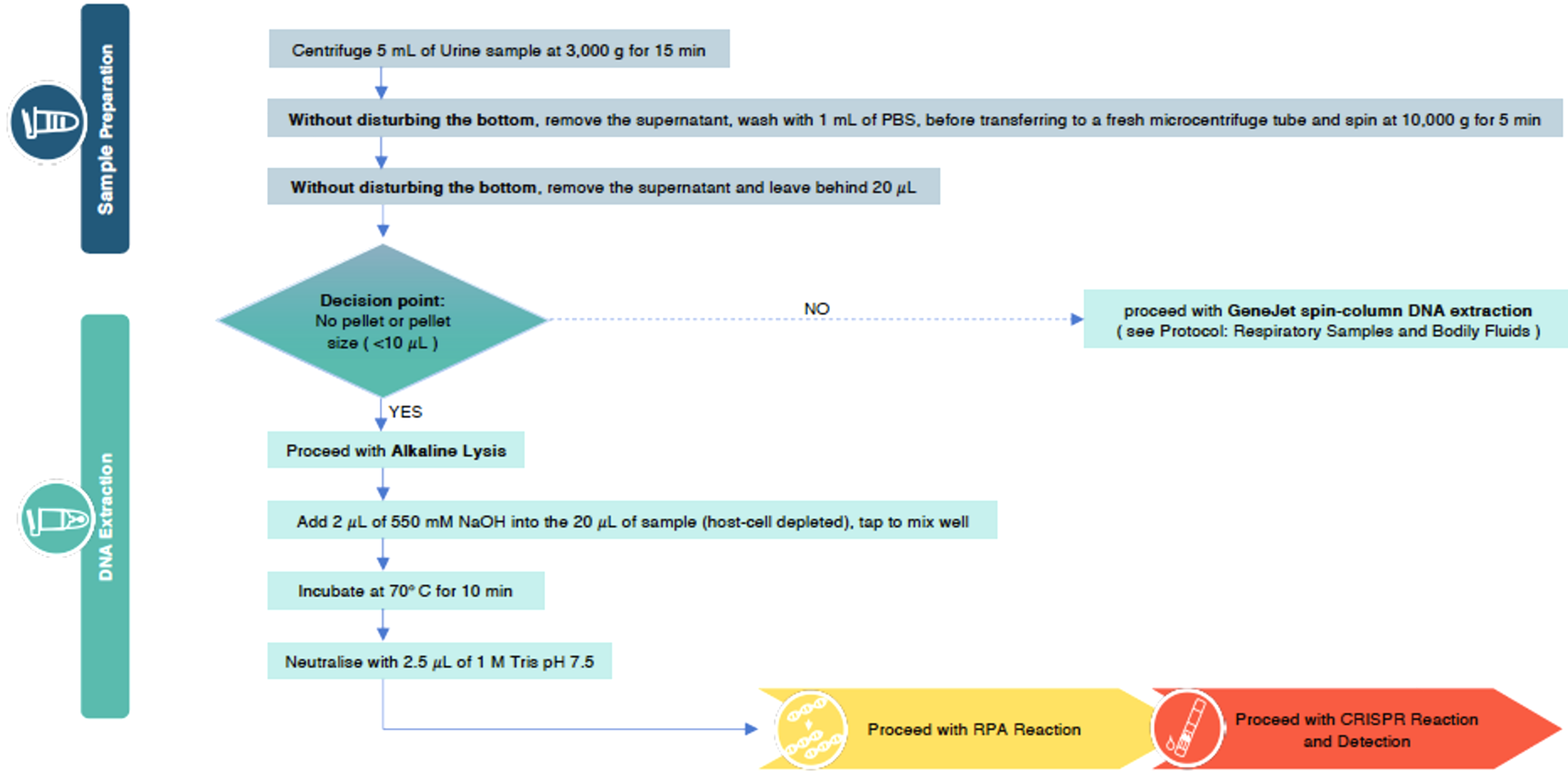

Supplementary Figure S5. CRISPR-BP34 assay flowchart for bodily fluids (such as sputum or pus)

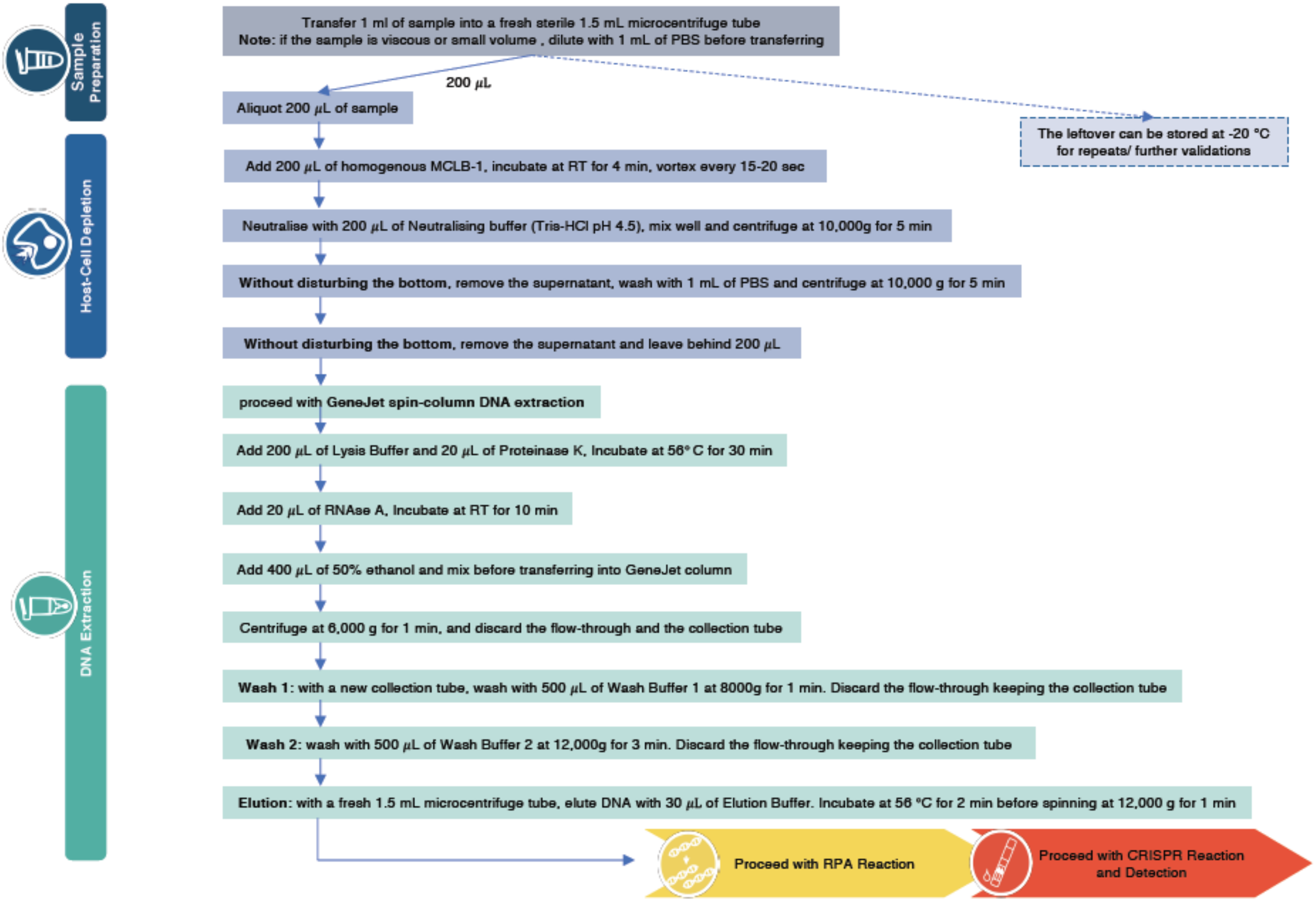

Supplementary Figure S6. RPA reaction assay flowchart

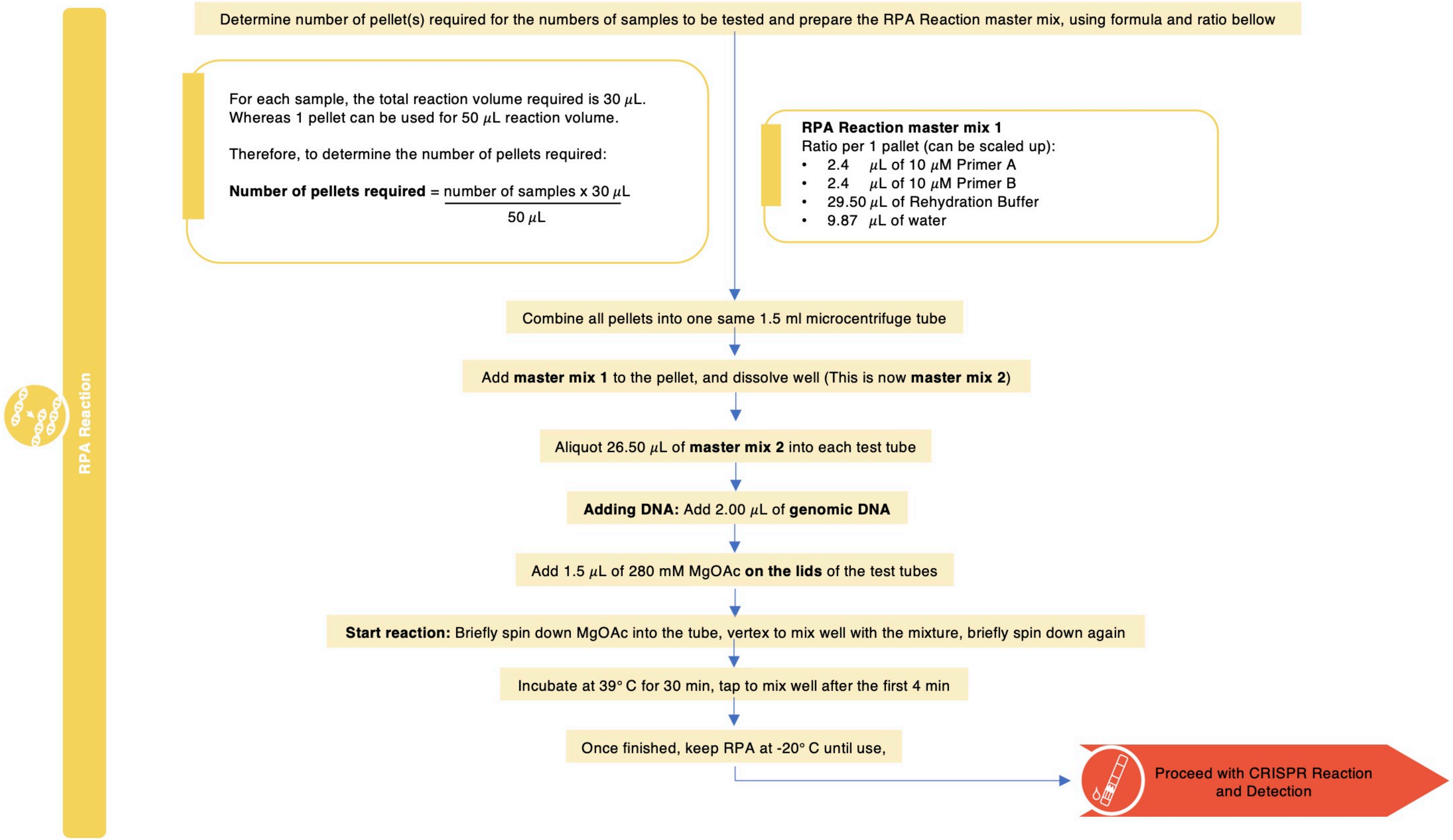

Supplementary Figure S7. CRISPR reaction and dipstick detection assay flowchart

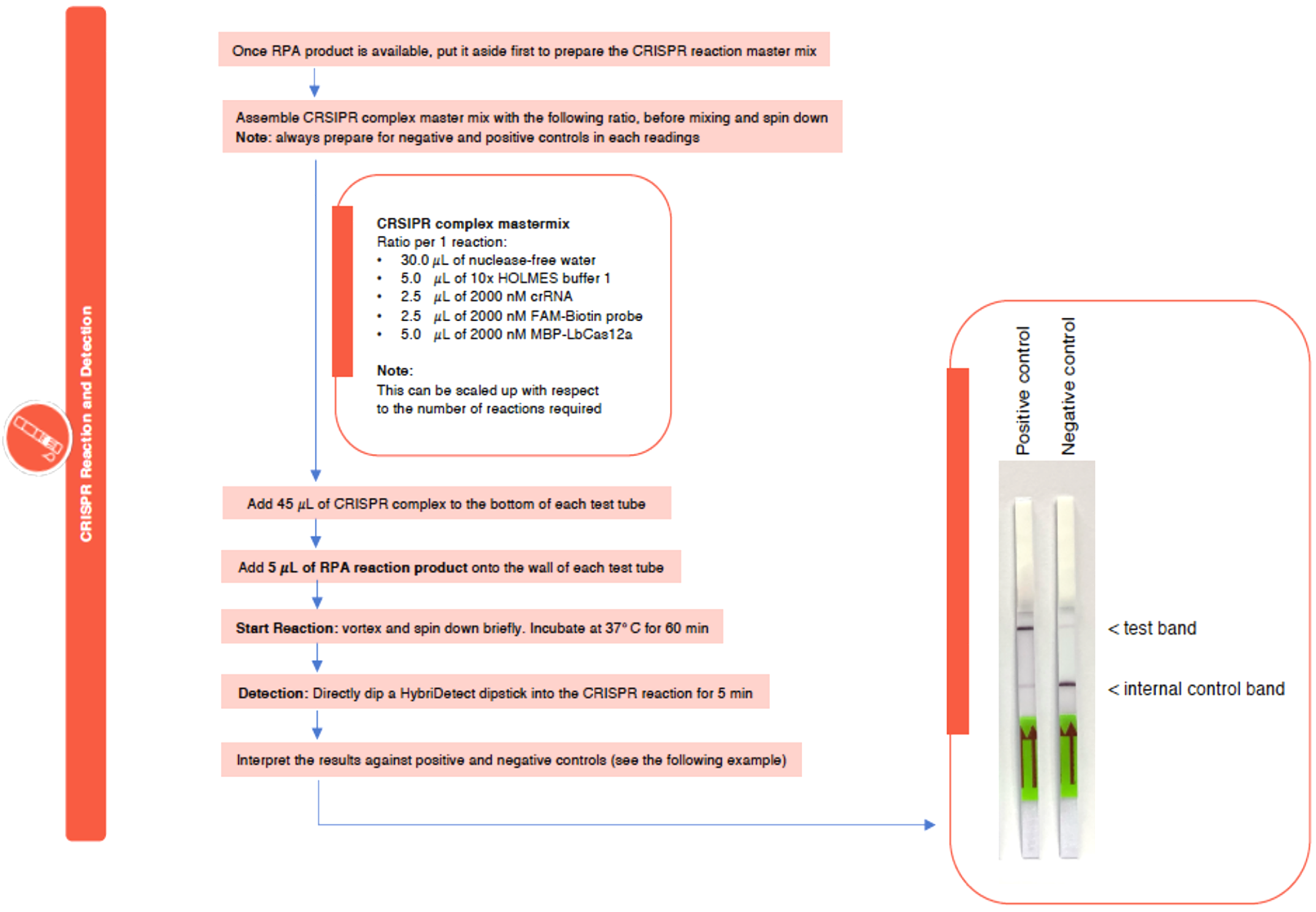

Supplementary Figure S8. Kaplan-Meier curves of demographic status and antibiotics prescription associated with melioidosis mortality post admission date

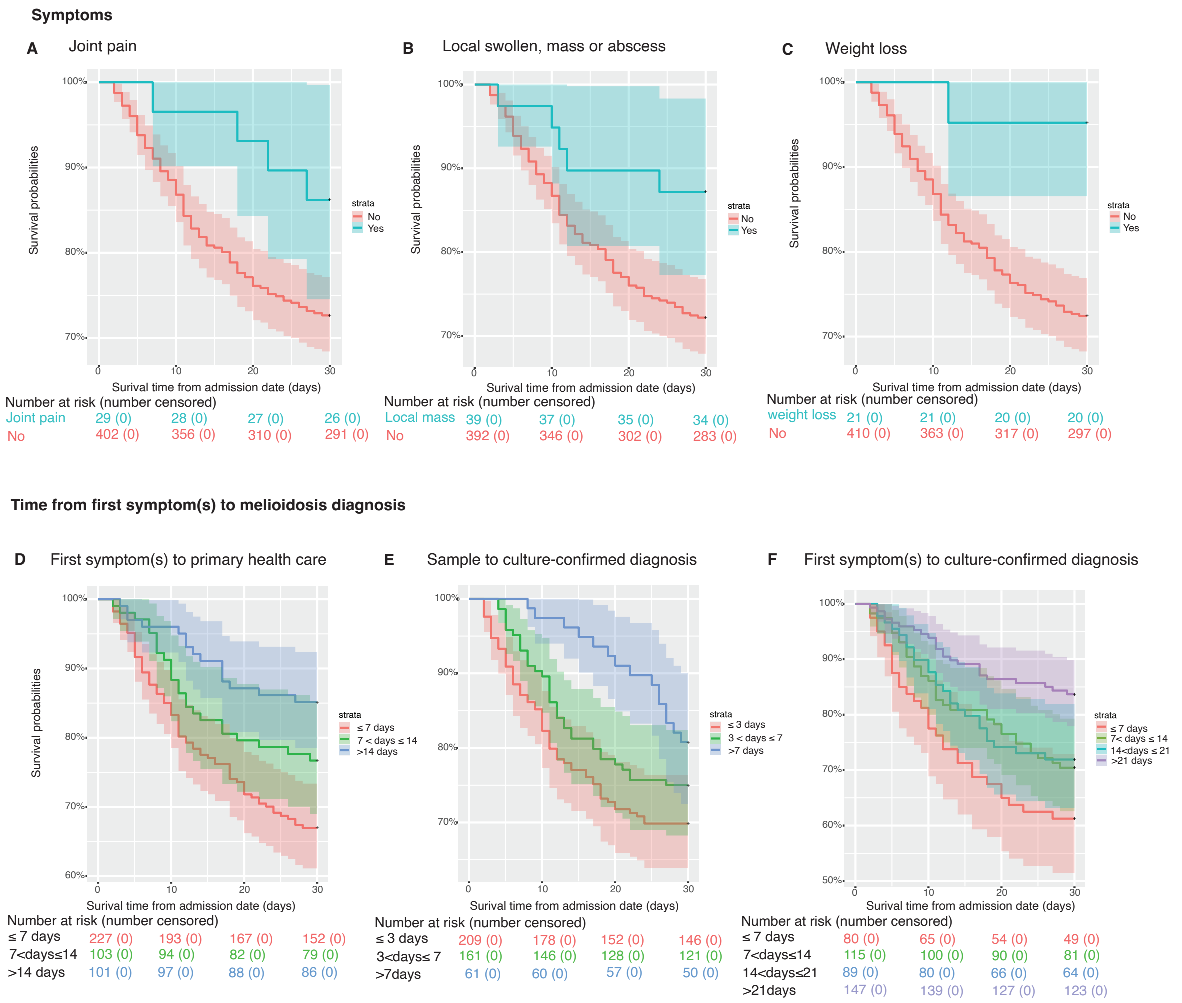

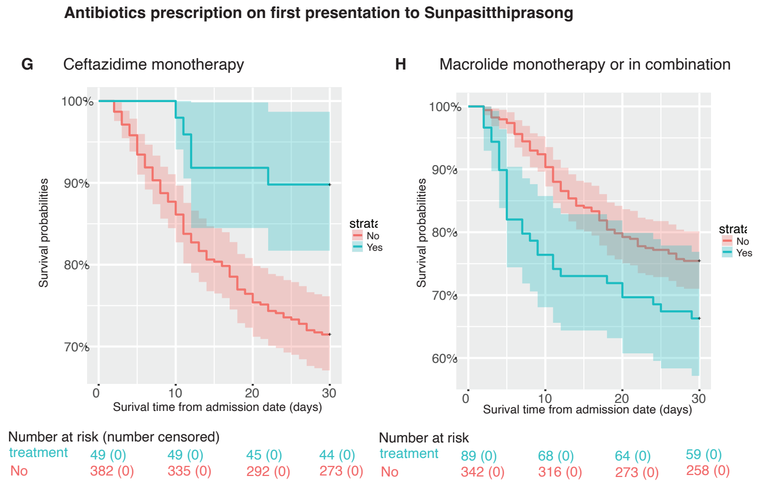

Localised symptoms (A and B) correlate with lower mortality as reported earlier[3,4,24]. Shorter symptom onset to healthcare seeking (D), faster positive culture conversion (E), and shorter overall symptom-to-culture diagnosis (F) highlight higher mortality linked to acute disease and likely elevated bacterial load. Ceftazidime monotherapy (G) exhibits reduced mortality per clinical trial result[25], while administering macrolide antibiotic (H) to which the bacterium is already resistant[26] associates with higher mortality.

Supplementary Figure S9. Time taken for the culture and the CRISPR-BP34 approaches, and case examples

A to **D** highlighted selected cases (4 of 20 melioidosis cases) where both multiple sample types and multiple specimens per sample type were available (vertical axis). The horizontal axis represents time in days from the date of admission or referral to Sunpasitthiprasong hospital. Circles mark the dates when samples were taken, while rectangles and triangles present the time when the culture-confirmed results and the CRISPR-BP34 results arrived, respectively. For both culture- and CRISPR-BP34 approaches; black denotes negative result, grey highlights detection of other bacterial species than *B. pseudomallei*, while pink (hemoculture), orange (urine), purple (respiratory secretion) and blue (pus, tissue, and other body fluids) present *B. pseudomallei* positive result from each sample type.

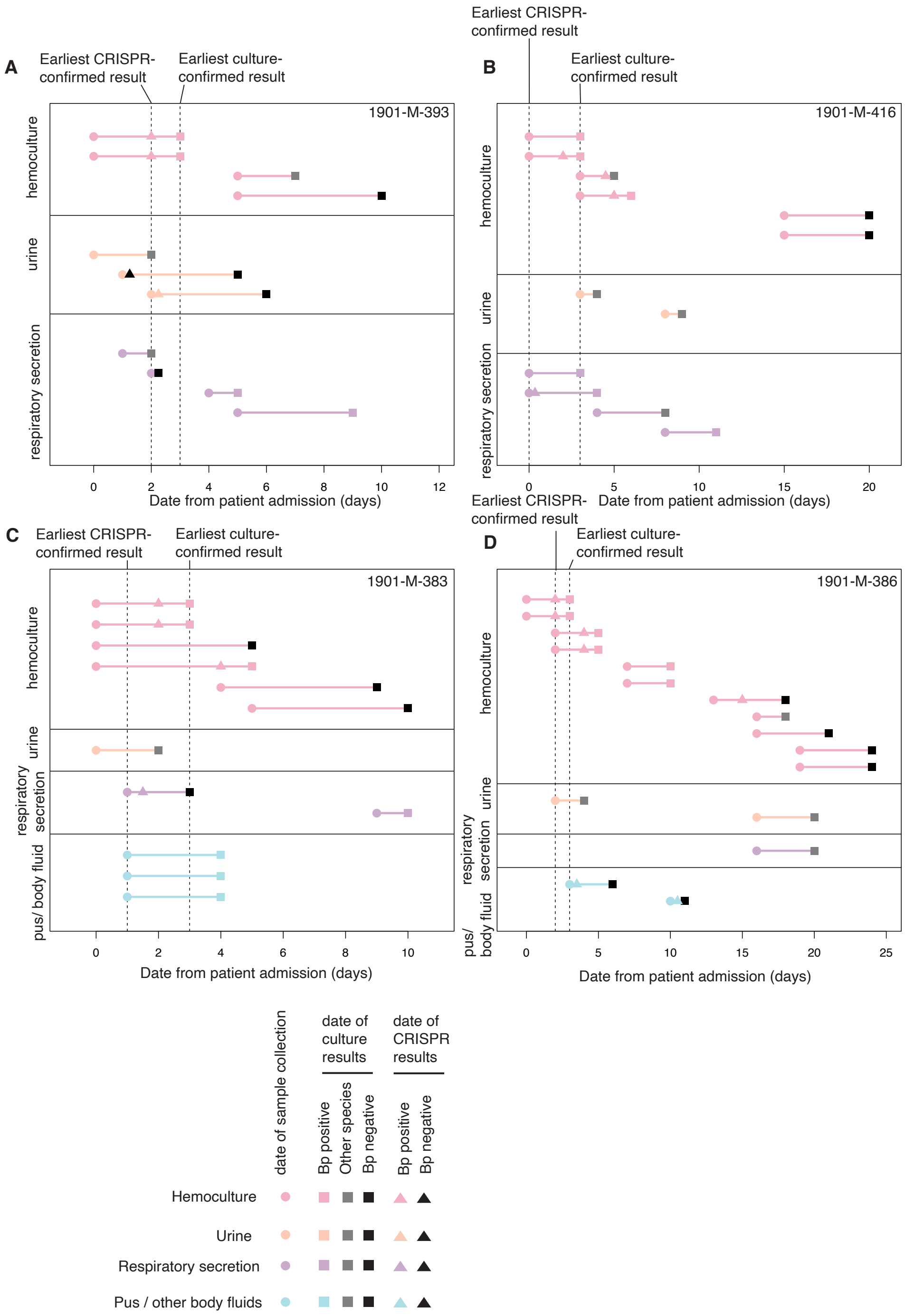

Supplementary Figure S10. Comparison of qPCR cycle threshold (ct scores) between culture positive and negative samples.

Scatter boxplots summarise the number of ct scores obtained from qPCR performed to validate culture negative but CRISPR-BP34 positive samples. Each individual dot represents a single test while each boxplot highlights the first quartile, median, and third quartile, respectively. Culture positive (hemoculture) samples were used as a positive control (left). Blank water was used as negative control (results not shown) Hemoculture (pink), urine (orange), respiratory secretion (purple) and pus (blue) with culture negative but CRISPR-BP34 positive results all show significantly higher ct scores. This suggests the presence of *B. pseudomallei* at low bacterial loads which are possibly under the culture limit of detection.

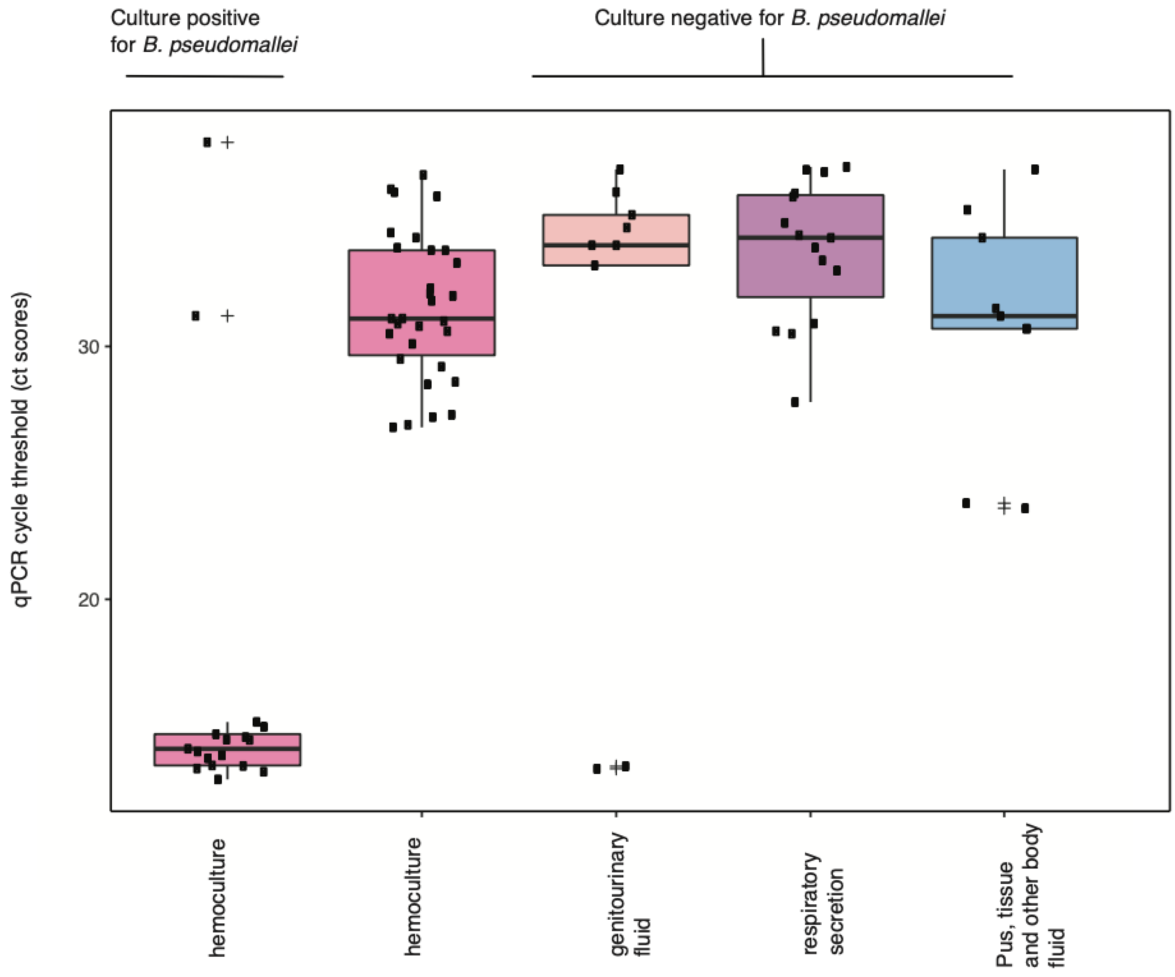

Supplementary Figure S11. Symptoms and types of melioidosis reported in the cohort.

An upset plot summarises the clinical manifestations and their intersecting sets of melioidosis patients admitted or referred to Sunpasitthiprasong hospital between October 2019 and December 2022 and their intersecting sets. Major eported symptoms ranged from fever, cough, dyspnea, GI disturbance, urinary symptoms, joint pain, muscle pain, local mass, swollen or abscesses, and weight loss.

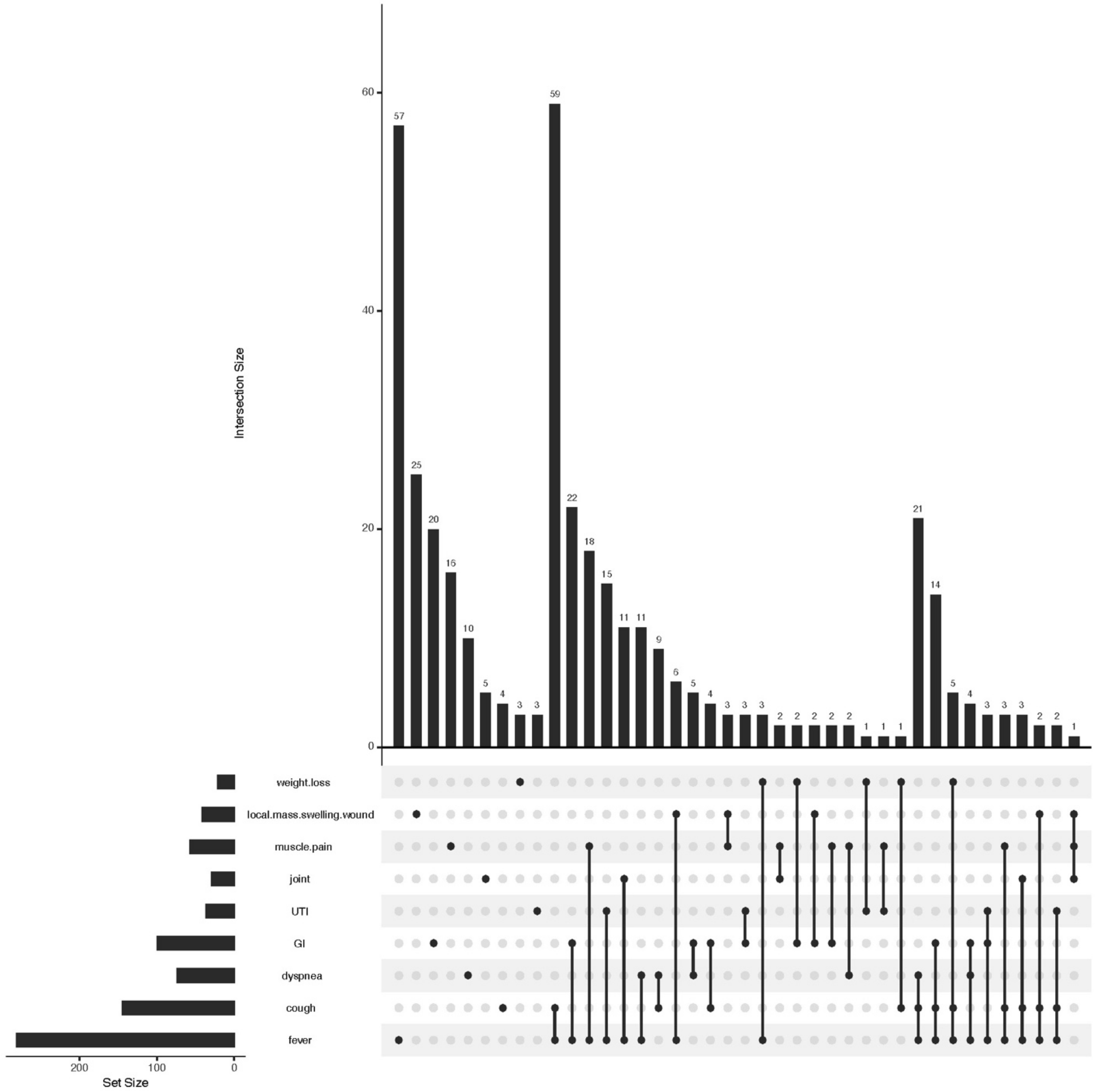

Supplementary Figure S12. Other pathogens reported in non-melioidosis controls

A pie-chart illustrates the species identified by Sunpasitthiprasong Hospital Central Lab as causative agents in our non-melioidosis patients. Species with higher prevalence were tested for potential CRISPR-BP34 cross-contamination *in vitro* as described in Wongpalee *et al.* 2022[7]

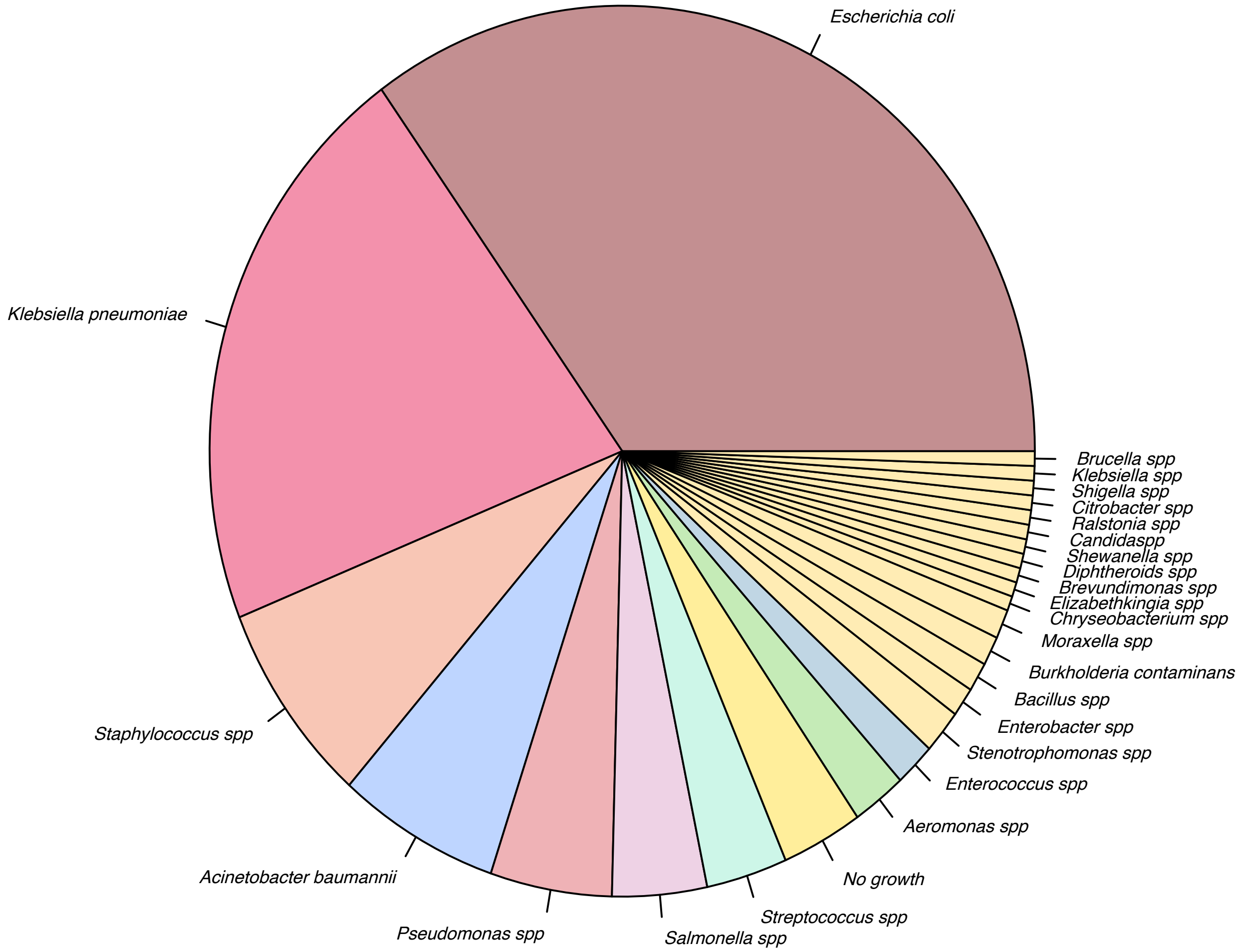

SUPPLEMENTARY TABLES

Supplementary Table S1. A list of primers and oligos used in the study

Name	Sequences	Usage	Reference
148	CAGCATATCATTGTCCGGCGCGAACCA TCAAGCTA	RPA primer for crBP34	[7]
149	AACTTTTCATTTTCCTGTCAATTCGACT GACCATC	RPA primer for crBP34	[7]
167	TAATACGACTCACTATAGGGTAATTTC TACTAAGTGTAGATACTACATACCCAC TATTCAGAAAGGAA	Annealing to DNA oligo 168 to create a T7 transcription template for crBP34 crRNA	[7]
168	TTCCTTTCTGAATAGTGGGTATGTAGT ATCTACACTTAGTAGAAATTACCCTAT AGTGAGTCGTATTA	Annealing to DNA oligo 167 to create a T7 transcription template for crBP34 crRNA	[7]
192	CGTCTCTATACTGTCGAGCAATCG	qPCR primer targeting orf2 within putative type III secretion system cluster 1 (TTS1) of *B. pseudomallei*	[27–29]
193	CGTGCACACCGGTCAGTATC	qPCR primer targeting orf2 within putative type III secretion system cluster 1 (TTS1) of *B. pseudomallei*	[27–29]
198	ATGCGACTCCTGCATTAGGAA	Amplifying the DNA insert from the genome of *E. coli* NEB 10-beta	This study
199	TTCGATTATGCGGCCGTGTA	Amplifying the DNA insert from the genome of *E. coli* NEB 10-beta	This study
200	AAGGATCCATGGCAGCATATCATTGTC CGGCG	Cloning of the RPA amplicon into pACYCDuet-1	This study
201	AAGGATCCATGGAACTTTTCATTTTCC TGTCAATT	Cloning of the RPA amplicon into pACYCDuet-1 plasmid	This study
217	TATGGAAACCGTCGATATTCAGC	Amplifying ~500-bp DNA from the lacZ locus and utilizing it as the upstream homology arm	This study
218	TTCCTAATGCAGGAGTCGCATAAGTGC ACGGCAGATACACTTG	Amplifying ~500-bp DNA from the lacZ locus and utilizing it as the upstream homology arm	This study
219	GAAGTGATCTTCCGTCACAGGGATCAT CGGTCAGACGATTCATT	Amplifying ~500-bp DNA from the lacZ locus and utilizing it as the downstream homology arm	This study
220	CGAGTTGCGTGACTACCTAC	DNA from the lacZ locus and utilizing it as the downstream homology arm	This study
221	TTATGCGACTCCTGCATTAGGAA	Amplifying RPA amplicon and Chloramphenicol resistance gene from pACYCDuet-1 plasmid	This study
222	CCTGTGACGGAAGATCACTTC	Amplifying RPA amplicon and Chloramphenicol resistance gene from pACYCDuet-1 plasmid	This study
270	AACACTGACAAGTGGCCCTATGGA	qPCR primer targeting orf11 (BPSS1386) of *B. pseudomallei*	[30]

Name	Sequences	Usage	Reference
271	TCCGATCGGTTTCGAATAACGGGT	qPCR primer targeting orf11 (BPSS1386) of *B. pseudomallei*	[30]
274	CTTCGGACCGCCTCGAACT	qPCR primer targeting crBP36 locus (BPSS1386 and intergenic region) of *B. pseudomallei*	This study
275	GCGAGGGTGTCGAACTGATGT	qPCR primer targeting crBP36 locus (BPSS1386 and intergenic region) of *B. pseudomallei*	This study
FAM-biotin probe	/56-FAM/TTATT/3Bio/	Collateral probe	[7,31]

Supplementary Table S2 Cox regression analysis evaluating demographic status and antibiotics prescribed on first day of admission associated with mortality

Univariable cox regression analyses were conducted individually for each variable. Additionally, a multivariate Cox regression analysis was performed on eight variables that exhibited significant associations in the univariate Cox regression analysis with prior evidence from the literature[3,4,24,25] indicating their connection to melioidosis mortality. The significance threshold was set at 0.05. * and ** denotes significance by univariate and multivariate analyses, respectively. Please note that the analysis was performed for variable with at least 5 events per variables.

Variables		Univariable cox regression			Multivariable cox regression		
		Coefficient	HR (95% CI)	p-val	Coefficient	HR (95% CI)	p-val
**Age (in years)**		0·0084	1·01 (0·99 - 1·02)	0·25	N/A	N/A	N/A
**Sex**	Female	0 (ref)	1 (ref)				
	Male	0·04	1.04 (0·69 - 1·55)	0·86	N/A	N/A	N/A
**Occupation**							
Agriculture	No	0 (ref)	1 (ref)				
	Yes	-0·05	0.95 (0·65 - 1·40)	0·80	N/A	N/A	N/A
Private sector	No	0 (ref)	1 (ref)				
	Yes	0·061	1·06 (0·66 - 1·71)	0·80	N/A	N/A	N/A
Homemaker	No	0 (ref)	1 (ref)				
	Yes	0·078	1·08 (0·63 - 1·86)	0·78	N/A	N/A	N/A
Monk	No	0 (ref)	1 (ref)				
	Yes	-0·0099)	0·99 (0·24 - 4·01)	0·99	N/A	N/A	N/A
Police and Military	No	0 (ref)	1 (ref)				
	Yes	0·20	1·23 (0·30 - 4·96)	0·78	N/A	N/A	N/A
**Travel distance (in km)**		0·0024	1·002(1.00 -1·007)	0·31	N/A	N/A	N/A
**Symptoms**							
Fever	No	0 (ref)	1 (ref)				
	Yes	0·047	1·05 (0·71 - 1·54)	0·81	N/A	N/A	N/A
Cough	No	0 (ref)	1 (ref)				
	Yes	0·31	1·36 (0·93-1·99)	0·11	N/A	N/A	N/A
Dyspnea	No	0 (ref)	1 (ref)				
	Yes	0·24	1·27 (0·80-2·02)	0·33	N/A	N/A	N/A
GI disturbance	No	0 (ref)	1 (ref)				
	Yes	0·36	1·43 (0·95-2·15)	0·097	N/A	N/A	N/A
Urinary symptoms	No	0 (ref)	1 (ref)				
	Yes	0·31	1·36 (0·75-2·48)	0·33	N/A	N/A	N/A
Joint pain*	No	0 (ref)	1 (ref)				

	Yes	-1·10	0·33 (0·11-1·05)	0·024	-0·77	0·46 (0·17-1·26)	0·13
Muscle pain	No	0 (ref)	1 (ref)				
	Yes	-0·22	0·80 (0·45-1·43)	0·44	N/A	N/A	N/A
Local swollen, mass or abscess*	No	0 (ref)	1 (ref)				
	Yes	-0·86	0·42 (0·17-1·03)	0·03	-0·82	0·44 (0·18-1·10)	0·079
Weight loss*	No	0 (ref)	1 (ref)				
	Yes	-1·89	0·15 (0·021-1·08)	0·0073	-1·84	0·16 (0·021-1·18)	0·073
**Time to melioidosis diagnosis in days**							
Symptom onset to approaching healthcare*		-0·018	0·98 (0·97 - 0·99)	0·0025	0·031	1·03 (0·98-1·09)	0·26
Refer system		-0·028	0·97 (0·93 - 1·02)	0·20	N/A	N/A	N/A
Sample to culture-diagnosis*		-0·050	0·95 (0·90 - 1·002)	0·033	-0·026	0·97 (0·90-1·05)	0·502
Symptom onset to culture-diagnosis*		-0·020	0·98 (0·97 - 0·99)	0·00042	-0·038	0·96 (0·92-1·01)	0·13
**Antibiotics prescribed on first presentation to Sunpasitthiprasong**							
ceftazidime alone**	No	0 (ref)	1 (ref)				
	Yes	-1·15	0·32 (0·13 - 0·79)	0·0026	-1·011	0·36 (0·15-0·89)	0·029
carbapenem alone	No	0 (ref)	1 (ref)				
	Yes	0·29	1·34 (0·80 - 2·24)	0·28	N/A	N/A	N/A
ceftazidime or carbapenem or both	No	0 (ref)	1 (ref)				
	Yes	-0·27	0·76 (0·49 - 1·20)	0·22	N/A	N/A	N/A
ceftazidime or carbapenem but with other antibiotics	No	0 (ref)	1 (ref)				
	Yes	0·18	1·20 (0·80 - 1·81)	0·38	N/A	N/A	N/A
ceftriaxone alone or in combination with other antibiotics	No	0 (ref)	1 (ref)				
	Yes	0·098	1·10 (0·72 - 1·69)	0·66	N/A	N/A	N/A
penicillin alone or in combination with other antibiotics	No	0 (ref)	1 (ref)				
	Yes	-0·24	0·79 (0·41 - 1·51)	0·46	N/A	N/A	N/A
lincosamide alone or in combination with other antibiotics	No	0 (ref)	1 (ref)				
	Yes	0·28	1·34 (0·80 - 2·24)	0·284	N/A	N/A	N/A
macrolide alone or in combination with other antibiotics*	No	0 (ref)	1 (ref)				
	Yes	0·45	1·56 (1·03 - 2·37)	0·043	0·36	1·44 (0·94-2·19)	0·093
nitroimidazole alone or in combination with other antibiotics	No	0 (ref)	1 (ref)				
	Yes	0·30	1·35 (0·77 - 2·36)	0·31	N/A	N/A	N/A

Supplementary Table S3. Clinical sensitivity and specificity of culture and CRISPR-based methods

Sample types	Disease status		Culture-based approach		CRISPR-BP34		McNemar test between paired data
	melioidosis cases	non-melioidosis controls	Sensitivity (Percent, 95% CI)	Specificity (Percent, 95% CI)	Sensitivity (Percent, 95% CI)	Specificity (Percent, 95% CI)	
Overall*	114	216	76 of 114 (66·7%, CI 57·2 – 75·2%)	216 of 216 (100%, CI 98·3 – 100·0%	106 of 114 (93·0%, CI 86·6 – 96·9%)	209 of 216 (96·8%, CI 93·4 – 98·7%)	<0·0001
Hemoculture	41	74	24 of 41 (58·5%, CI 42·1 – 73·7%)	74 of 74 100%, CI 95·1 – 100·0%)	41 of 41 (100·0%, CI 91·40 – 100·0%)	70 of 74 (94·6%, CI 86·7 – 98·5%)	<0·0001
Genitourinary fluid	27	71	14 of 27 (51·9%, CI 32·0 – 73·3%)	71 of 71 (100%, CI 94·9 – 100·0%)	19 of 27 (70·4%, CI 49·8 – 86·3%)	70 of 71 (98·6%, CI 92·4 – 100%)	0·04
Respiratory secretion	52	54	44 of 52 (84·6%, CI 71·9 – 93·1%)	54 of 54 (100%, CI 93·4 – 100·0%)	49 of 52 (94·2%, CI 84·1-98·8%)	53 of 54 (98·2%, CI 90·1-99·6%)	0·15
Pus, tissue and other body fluids	15	30	11 of 15 (73·3%, CI 44·9-92·2%)	30 of 30 (100%, CI 88·4 -100·0%)	15 of 15 (100%, CI 78·2 – 100·0%)	29 of 30 (96·67%, CI 82·8-99·9%)	0·15

*For patients with multiple samples taken, only the first sample from each patient was retained for statistical analysis.

Supplementary Table S4. Stages of CRISPR-BP34 development by WHO ASSURED guideline

Test parameters	CRISPR-BP34	Further development
Diagnostic target	DNA	DNA
Test format	Lateral flow dipstick	Lateral flow dipstick
End-user	Provincial (specialists)	District (technicians) or Primary care
**R**eal-time connectivity	Human input to interpret results is still required	Human input to interpret results is still required
**E**ase of specimen collection and processing	Dedicated DNA extraction step is still required	Dedicated DNA extraction step is still required
**A**ffordable (A reaction cost) (Staff cost per day)	~ 15 USD (laboratory scale) ~ 20.0 to 28.7 staff/day	<10 USD (pre-industrial scale) ~ 20.0 to 28.7 staff/day
**S**ensitivity (%)	93.0	93.0 or less to compromise one pot reaction
**S**pecificity (%)	96.8	96.8
**U**ser-friendly	Multiple processing steps are still required	Simplified processing with pre-measured freeze-dried pellet
**R**apid and robust	~4 hours (including DNA extraction and handling time)	<3.5 hours (one-pot RPA and CRISPR reaction)
**E**quipment-free	Heatblock and pipette are still needed	Heatblock and pipette are still needed
**D**eliverable	Cold shipment and refrigerator required	Shipment and storage can be performed at room temperature

SUPPLEMENTARY REFERENCES

1 Angchagun K, Boonklang P, Chomkatekaew C, *et al.* BurkHostGEN: a study protocol for evaluating variations in the Burkholderia pseudomallei and host genomes associated with melioidosis infection. *Wellcome Open Res* 2023. DOI:10.12688/wellcomeopenres.19809.1.

2 Limmathurotsakul D, Peacock SJ. Melioidosis: a clinical overview. *Br Med Bull* 2011; **99**: 125–39.

3 Hantrakun V, Kongyu S, Klaytong P, *et al.* Clinical Epidemiology of 7126 Melioidosis Patients in Thailand and the Implications for a National Notifiable Diseases Surveillance System. *Open Forum Infect Dis* 2019; **6**: ofz498.

4 Nathan S, Chieng S, Kingsley P, *et al.* Melioidosis in Malaysia: Incidence, Clinical Challenges, and Advances in Understanding Pathogenesis. *TropicalMed* 2018; **3**: 25.

5 WHO. Guidelines for the collection of clinical specimens during field investigation of outbreaks. 2000. https://apps.who.int/iris/handle/10665/66348. accessed date 1 October 2019

6 Department of Disease Control, Ministry of Public Health Thailand. Guideline of Melioidosis, 1st edn. 2021 http://klb.ddc.moph.go.th/dataentry/handbook/form/129.

7 Wongpalee SP, Thananchai H, Chewapreecha C, *et al.* Highly specific and sensitive detection of Burkholderia pseudomallei genomic DNA by CRISPR-Cas12a. *PLoS Negl Trop Dis* 2022; **16**: e0010659.

8 Datsenko KA, Wanner BL. One-step inactivation of chromosomal genes in Escherichia coli K-12 using PCR products. *Proc Natl Acad Sci U S A* 2000; **97**: 6640–5.

9 Lesic B, Rahme LG. Use of the lambda Red recombinase system to rapidly generate mutants in Pseudomonas aeruginosa. *BMC Mol Biol* 2008; **9**: 20.

10 Serra-Moreno R, Acosta S, Hernalsteens JP, Jofre J, Muniesa M. Use of the lambda Red recombinase system to produce recombinant prophages carrying antibiotic resistance genes. *BMC Mol Biol* 2006; **7**: 31.

11 Kobayashi A, Sano D, Taniuchi A, Ishii S, Okabe S. Use of a genetically-engineered Escherichia coli strain as a sample process control for quantification of the host-specific bacterial genetic markers. *Appl Microbiol Biotechnol* 2013; **97**: 9165–73.

12 H. Abdelhai M. Comparative Study of Rapid DNA Extraction Methods of Pathogenic Bacteria. *BIO* 2016; **4**: 1.

13 Pilecky M, Schildberger A, Orth-Höller D, Weber V. Pathogen enrichment from human whole blood for the diagnosis of bloodstream infection: Prospects and limitations. *Diagn Microbiol Infect Dis* 2019; **94**: 7–14.

14 Mancini N, Carletti S, Ghidoli N, Cichero P, Burioni R, Clementi M. The Era of Molecular and Other Non-Culture-Based Methods in Diagnosis of Sepsis. *Clin Microbiol Rev* 2010; **23**: 235–51.

15 Döring G, Unertl K, Heininger A. Validation criteria for nucleic acid amplification techniques for bacterial infections. *Clin Chem Lab Med* 2008; **46**: 909–18.

16 Handschur M, Karlic H, Hertel C, Pfeilstöcker M, Haslberger AG. Preanalytic removal of human DNA eliminates false signals in general 16S rDNA PCR monitoring of bacterial pathogens in blood. *Comp Immunol Microbiol Infect Dis* 2009; **32**: 207–19.

17 Trung NT, Hien TTT, Huyen TTT, *et al.* Enrichment of bacterial DNA for the diagnosis of blood stream infections. *BMC Infect Dis* 2016; **16**: 235.

18 Van Meerbergen B, Piciu O, Gill R, *et al.* Selective lysis of cells. https://patents.google.com/patent/WO2011070507A1/en (accessed April 5, 2022).

19 Lv H, Wang J, Zhang J, *et al.* Definition of CRISPR Cas12a Trans-Cleavage Units to Facilitate CRISPR Diagnostics. *Front Microbiol* 2021; **12**: 766464.

20 Wuthiekanun V, Limmathurotsakul D, Wongsuvan G, *et al.* Quantitation of B. Pseudomallei in clinical samples. *Am J Trop Med Hyg* 2007; **77**: 812–3.

21 Fairley L, Smith S, Maisrikrod S, Henning L. Systematic review and meta-analysis of diagnostic tests for diagnosis of melioidosis. *Acta Trop* 2021; **214**: 105784.

22 Land KJ, Boeras DI, Chen X-S, Ramsay AR, Peeling RW. REASSURED diagnostics to inform disease control strategies, strengthen health systems and improve patient outcomes. *Nat Microbiol* 2019; **4**: 46–54.

23 Hu F, Liu Y, Zhao S, *et al.* A one-pot CRISPR/Cas13a-based contamination-free biosensor for low-cost and rapid nucleic acid diagnostics. *Biosens Bioelectron* 2022; **202**: 113994.

24 Chien JM-F, Saffari SE, Tan A-L, Tan T-T. Factors affecting clinical outcomes in the management of melioidosis in Singapore: a 16-year case series. *BMC Infect Dis* 2018; **18**: 482.

25 Chierakul W, Anunnatsiri S, Short JM, *et al.* Two Randomized Controlled Trials of Ceftazidime Alone versus Ceftazidime in Combination with Trimethoprim-Sulfamethoxazole for the Treatment of Severe Melioidosis. *Clinical Infectious Diseases* 2005; **41**: 1105–13.

26 Simpson AJ, White NJ, Wuthiekanun V. Aminoglycoside and macrolide resistance in Burkholderia pseudomallei. *Antimicrob Agents Chemother* 1999; **43**: 2332.

27 Novak RT, Glass MB, Gee JE, *et al.* Development and Evaluation of a Real-Time PCR Assay Targeting the Type III Secretion System of *Burkholderia pseudomallei*. *J Clin Microbiol* 2006; **44**: 85–90.

28 Chantratita N, Meumann E, Thanwisai A, *et al.* Loop-mediated isothermal amplification method targeting the TTS1 gene cluster for detection of Burkholderia pseudomallei and diagnosis of melioidosis. *J Clin Microbiol* 2008; **46**: 568–73.

29 Kaestli M, Richardson LJ, Colman RE, *et al.* Comparison of TaqMan PCR assays for detection of the melioidosis agent Burkholderia pseudomallei in clinical specimens. *J Clin Microbiol* 2012; **50**: 2059–62.

30 Lowe W, March JK, Bunnell AJ, O'Neill KL, Robison RA. PCR-based Methodologies Used to Detect and Differentiate the Burkholderia pseudomallei complex: B. pseudomallei, B. mallei, and B. thailandensis. *Curr Issues Mol Biol* 2014; **16**: 23–54.

31 Nguyen LT, Smith BM, Jain PK. Enhancement of trans-cleavage activity of Cas12a with engineered crRNA enables amplified nucleic acid detection. *Nat Commun* 2020; **11**: 4906.
